# TRIM21 mediates the synergistic effect of Olaparib and Sorafenib by degrading BRCA1 through ubiquitination in TNBC

**DOI:** 10.1038/s41523-023-00588-1

**Published:** 2023-10-20

**Authors:** Ning Huang, Peng Li, Xiaolin Sun, Li Tong, Xinyi Dong, Xuemei Zhang, Jifeng Duan, Xia Sheng, Hong Xin

**Affiliations:** 1 Department of Pathology, Minhang Hospital & Department of Pharmacology, School of Pharmacy, Fudan University, Shanghai, China; 2 PharmaLegacy Laboratories Co., Ltd, Shengrong Road No.388, Zhangjiang High-tech Park, Pudong New Area, Shanghai, China

**Keywords:** Breast cancer, Cancer therapy

## Abstract

Triple-negative breast cancer (TNBC) is a heterogeneous and aggressive type of breast cancer with a poor prognosis and a high recurrence rate. Chemotherapy is still the mainstay of treatment for cancer patients without a genetic BRCA mutation, despite the approval of Olaparib, an inhibitor of the poly (ADP-ribose) polymerase (PARP) enzyme. Tripartite motif containing-21 (TRIM21) is one of the TRIM family members that has been investigated in various types of cancer. Here, we found that a low TRIM21 expression level was correlated with poor overall survival of TNBC patients. Knockout of TRIM21 promoted the proliferation of TNBC cells in vivo and in vitro, as well as migratory and invasive capabilities in vitro. Importantly, breast cancer susceptibility gene 1 (BRCA1) was identified as a ubiquitination substrate of TRIM21. It was confirmed that BRCA1 was upregulated after Olaparib treatment, which may explain the relative resistance of BRCA1-proficient TNBC cells to Olaparib. Moreover, Sorafenib, a standard treatment for hepatocellular carcinoma, increased the sensitivity of TNBC cells to Olaparib by promoting TRIM21-mediated ubiquitination degradation of BRCA1. Thus, a synergic effect of Olaparib and Sorafenib was found in vitro and in vivo. This combined treatment also aggravated DNA damage, cell cycle arrest, and apoptosis of TNBC cells. In summary, the findings verified the synergistic effect of Olaparib and Sorafenib and revealed TRIM21 as a potential target for TNBC therapy.

## Introduction

As the most common cancer diagnosed and the second leading cause of cancer mortality in women worldwide^1^, breast cancer is characterized by inter- and intra-tumoral heterogeneity. According to the histological stratification, which is based on the expression levels of the progesterone receptor (PR), estrogen receptor (ER), and human epidermal growth factor receptor 2 (HER2), Breast cancer can be classified into distinct subtypes: normal-like, HER2-enriched, luminal A, luminal B, and triple-negative breast cancer (TNBC or basal-like breast cancer)^2,3^. TNBC lacks the expression of PR, ER, and HER2^4^. TNBC accounts for 10–15% of breast cancer^5^. It represents the malignant type with high invasive capability, metastatic potential, relapse proneness, fewer treatment options, and a worse prognosis outcome^6^. Currently, systemic therapies, such as chemotherapy, are the main treatment for TNBC. Targeted therapies are still under development and have shown some success. Olaparib, an inhibitor of the poly (ADP-ribose) polymerase (PARP) enzyme, has been approved for the treatment of patients with deleterious or suspected deleterious germline BRCA-mutated (gBRCAm), HER2-negative metastatic breast cancer^7^. PARP inhibitors are designed to exploit synthetic lethality known as “loss of either of the two key genes is not fatal to eukaryotic cells, and inactivation of both genes leads to cell death”^8^. PARP is involved in the repair of single-strand breaks (SSBs) of DNA. PARP inhibition caused by PARP inhibitors alone is not fatal because DNA damage can be repaired by other DNA repair pathways, particularly homologous recombination (HR) repair. Since BRCA1/2 is a key protein in HR repair of double-strand breaks (DSBs)^9^, tumor cells with BRCA gene mutations or deficiency are selectively sensitive to PARP inhibitors^10,11^. The mutation prevalence rates of BRCA1 or BRCA2 vary from 0.5 to 34% in different race or ethnicity groups^12^. Only about 10% of TNBC patients are BRCA1 mutation carriers in America^13,14^. Despite the development of several available surgical and chemotherapies or targeted therapies, the prognosis for TNBC patients without BRCA1 mutation remains unsatisfactory.

Represented as one of the largest classes of E3 ubiquitin ligases, the tripartite motif (TRIM) family proteins comprised three types of domains: RING domain, B-box domain, and coiled-coil (CC) region. TRIM family is involved in a wide range of cellular signaling transduction pathways and various biological processes. Consequently, it contributes to tumorigenesis, cancer development, and therapeutical resistance, exhibiting oncogenic or tumor-suppressive functions in diverse cancer^15^. The tripartite motif containing-21 (TRIM21), known as Ro52/SSA, is a special member of the TRIM family. It serves as an intracellular atypical cytosolic Fc receptor with a remarkable antibody isotype specificity. The pivotal role of TRIM21 in E3 ubiquitin ligase. TRIM21 is ubiquitously expressed and has a complex series of substrates. TRIM21 was reported to be correlated with different autoimmune diseases, such as systemic lupus erythematosus^16,17^ and Alzheimer’s disease^18^. Importantly, TRIM21 has been extensively involved in cancer development^19^. In breast cancer, TRIM21 has been indicated as a tumor suppressor or reported to participate in the dysregulation of the oncogenic signaling pathway^20–22^. Nevertheless, these studies have not fully investigated the role of TRIM21 in TNBC and the therapeutic potential for patients without BRCA1 mutation.

In this study, we identified TRIM21 as a positive prognostic factor in TNBC. The knockout of TRIM21 promoted TNBC cell proliferation in vitro and in vivo, as well as migratory and invasive capabilities in vitro. We also identified BRCA1 as a downstream ubiquitination substrate of TRIM21. Moreover, it was demonstrated that Sorafenib could downregulate BRCA1 through TRIM21-mediated ubiquitination degradation. Overall, we verified that TRIM21 plays an important role in augmenting the synergistic effect of Sorafenib and Olaparib in treating BRCA1-proficient TNBC patients.

## Results

### Knockout of TRIM21 promoted the proliferative, migratory, and invasive capabilities of TNBC cells

**Fig. 1:**
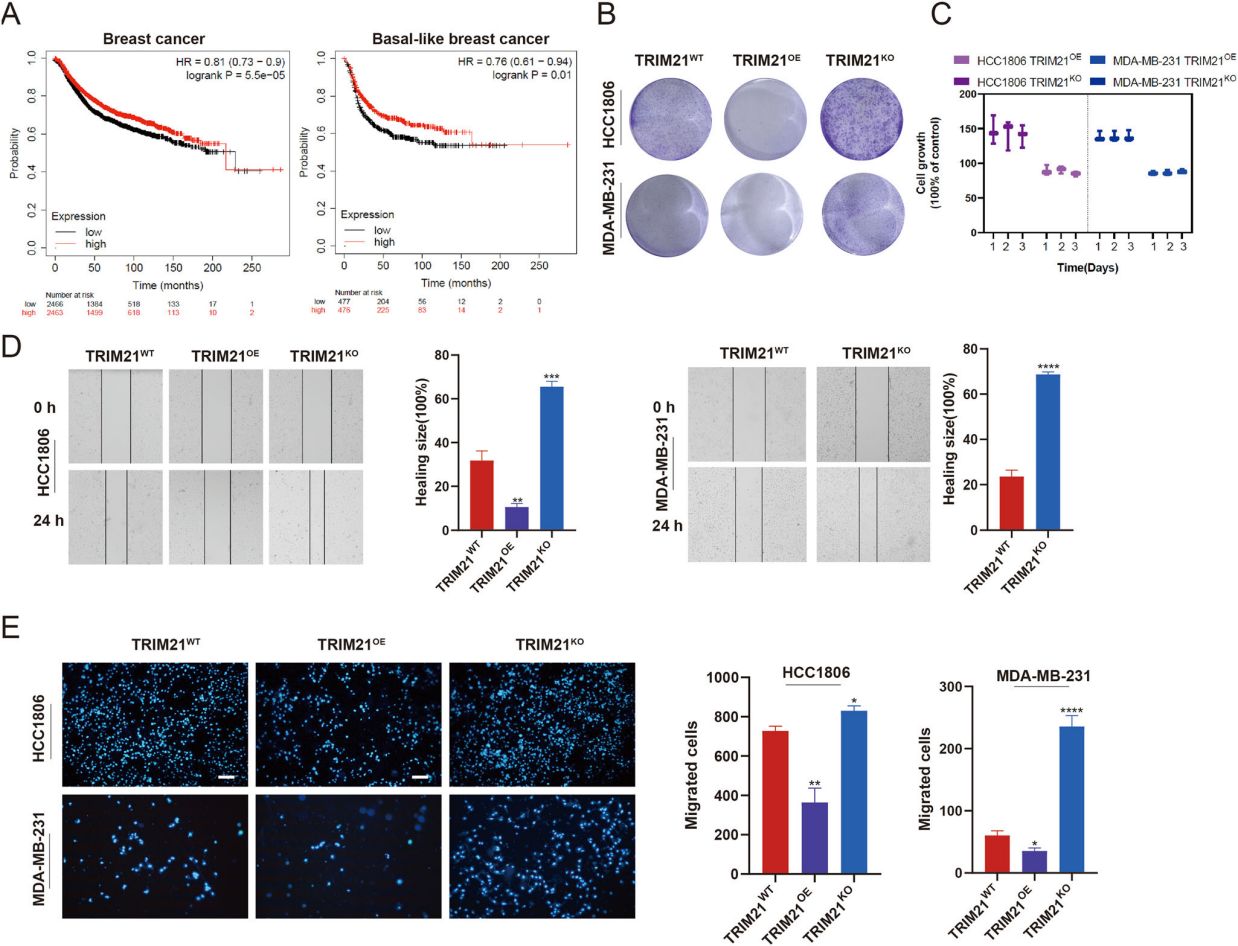
Knockout of TRIM21 promotes the proliferation, migration, and invasion capability of TNBC cells. **A** The correlation between the expression level of TRIM21 and relapse-free survival of breast cancer and basal-like breast cancer, respectively. **B** Colony formation assay and **C** CTG assay were performed to detect the proliferation capability of TRIM21^KO^ and TRIM21^OE^ TNBC cells. **D** The migration abilities were measured by wound-healing assay. The quantitative wound-healing trends obtained from wound size were illustrated. **E** The invasion abilities were indicated by transwell assay and presented as photofluorograms through DAPI staining. Data are shown as the mean ± SD of at least three independent experiments. Bar length: 100 μm. The significant level was identified by *P < 0.05, **P < 0.01, ***P < 0.001 and ****P < 0.0001 by one-way ANOVA.

The prognostic value of TRIM21 was assessed through online survival analysis with the Kaplan–Meier plotter tool by gene-chip data. An elevated TRIM21 mRNA expression level was associated with a longer relapse-free survival time in breast cancer and basal-like breast cancer (Fig. 1A) but no association in other subtype breast cancers (Supplementary Fig. 1). To investigate the role of TRIM21 in TNBC cells, TRIM21 knockout (TRIM21^KO^) and TRIM21 stably overexpressed (TRIM21^OE^) MDA-MB-231 and HCC1806 cell lines were constructed by lentiviral infection (Supplementary Fig. 1). By colony formation assay, it was found that the colony number of TRIM21^KO^ cells was significantly increased, while that of TRIM21^OE^ cells was reduced compared with the parental cells (Fig. 1B). The proliferation rate indicated by CTG assay (Fig. 1C) also proved that TRIM21 knockout enhanced the proliferation capability of TNBC cells. In addition, the migratory and invasive capabilities of TNBC cells were assessed by wound-healing assay (Fig. 1D) and transwell assay (Fig. 1E), respectively. Consistently, the knockout of TRIM21 increased the migratory and invasive capabilities of MDA-MB-231 and HCC1806 cells. TRIM21 overexpression showed the opposite effect. Collectively, these results are consistent with the previous studies that TRIM21 acts as a tumor suppressor in TNBC.

### TRIM21 mediated the ubiquitination degradation of BRCA1

**Fig. 2:**
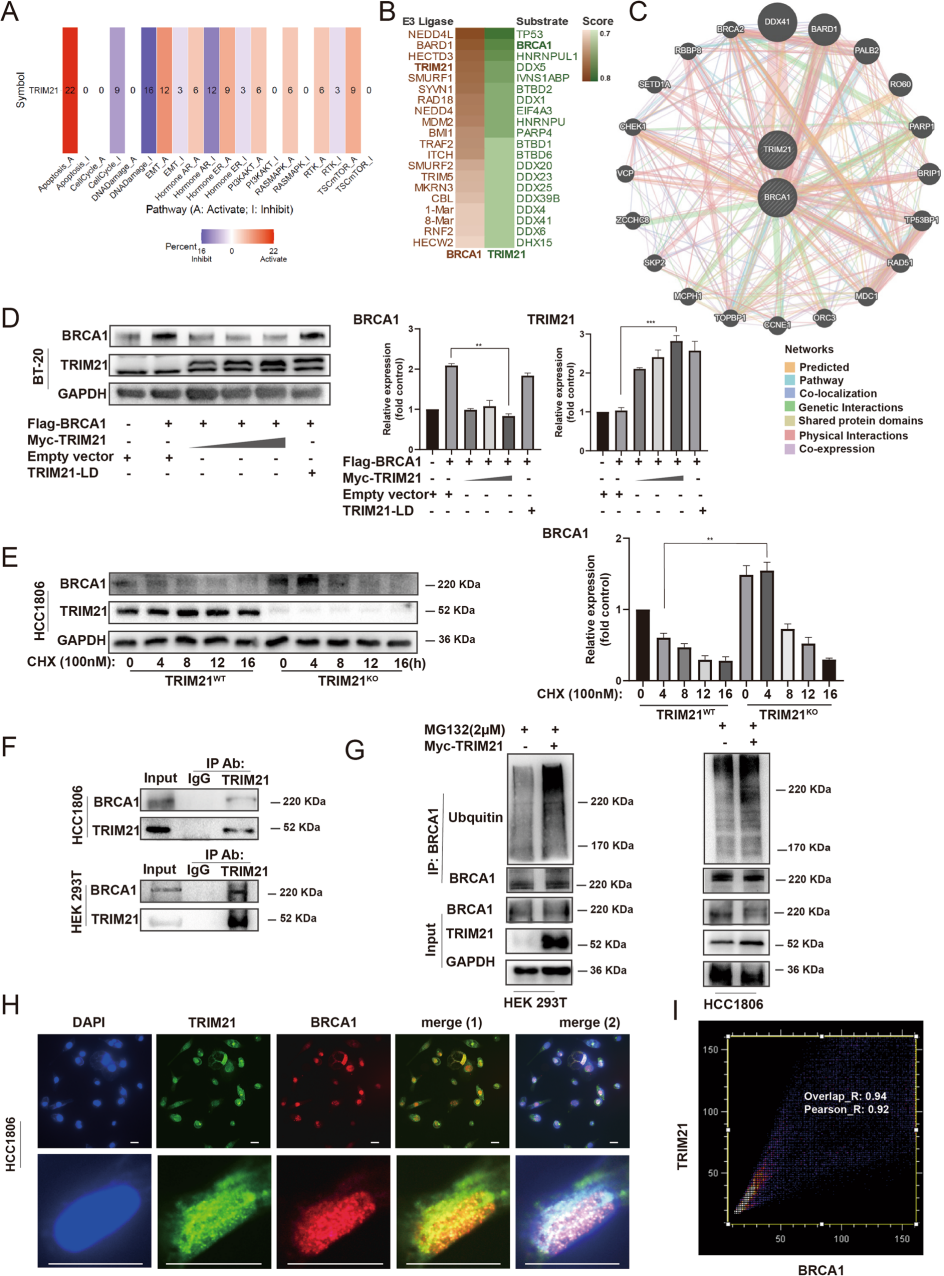
TRIM21 mediated the ubiquitination degradation of BRCA1. **A** A pan-cancer analysis between TRIM21 and signaling pathways by Gene Set Cancer Analysis. **B** Potential E3 ligases or substrates are predicted by using UbiBrowser. **C** TRIM21-BRCA1 Interaction analysis was performed in the GeneMANIA database. The specific interaction and predictive values are visible on (http://genemania.org/search/homo-sapiens/TRIM21/BRCA1). **D** The protein level was examined after transfecting with exogenous full-length BRCA1, TRIM21, and Ligase-dead TRIM21. The GAPDH expression is the loading control. **E** BRCA1 protein degradation rate in TRIM21^WT^ and TRIM21^KO^ cells. Data were shown as mean ± SD. The significant level was identified by *P < 0.05, **P < 0.01, ***P < 0.001 and ****P < 0.0001. One-way ANOVA analysis is used for (**D**) and (**E**). **F** The CO-IP results between TRIM21 and BRCA1. **G** 293T cells were transfected with Myc-TRIM21, and cell lysates were subjected to analysis by IP, followed by IB detection. **H** Immunofluorescent staining revealed the colocalization of TRIM21 and BRCA1 proteins in MDA-MB-231 cells. BRCA1 (red) and TRIM21 (green) and nuclear (blue). Bar length: 10 μm. **I** Colocalization analysis for immunofluorescent stained nuclear by ImageJ with colocalization plugin JACoP. *X*-axis: red pixel intensity (BRCA1). *Y*-axis: green pixel intensity (TRIM21). Overlap_R = 0.94, Pearson_R = 0.92 (calculations were based on data from three independent experiments, at least 15 nucleoli were analyzed).

To explore the potential pathways or molecules that TRIM21 may affect, pan-cancer analysis was conducted by gene set cancer analysis (Fig. 2A). The number of each frame type indicates the number of cancer types. TRIM21 showed a significant association with the inhibition of DNA damage response pathways among 16 types of cancer. Moreover, the possible substrates of TRIM21 and possible E3 ligase of BRCA1 were predicted using UbiBrowser (Fig. 2B). The results revealed that BRCA1 could be the substrate of TRIM21, and TRIM21 could be the possible E3 ligase of BRCA1. GeneMANIA online tool was used to identify regulatory networks of BRCA1 and TRIM21. Few proteins, including RAD51, are associated with the repair of DNA double-strand breaks (DSBs)^23^ and were found to be correlated with both TRIM21 and BRCA1 (Fig. 2C). To validate the predicted results, exogenous full-length TRIM21, BRCA1, and ligase-dead TRIM21 were transfected into the TNBC cells. The BRCA1 protein level exhibited a decrease in the TRIM21 overexpression group, whereas the TRIM21-LD group showed no significant change (Fig. 2D) The endogenous BRCA1 protein level was also decreased after transfecting with exogenous TRIM21 (Supplementary Fig. 2C). The cycloheximide (CHX) chase experiment was then conducted to determine the turnover of BRCA1, TRIM21 knockout appears to increase the stability and inhibit the turnover of the BRCA1 (Fig. 2E). To examine whether TRIM21 could directly bind to BRCA1, a co-immunoprecipitation (Co-IP) experiment was performed on HEK293T and HCC1806 cells to assess the interaction between TRIM21 and BRCA1. Endogenous physical interaction between the two proteins was confirmed by the Co-IP assay. However, because TRIM21 may be pulled down in an unspecific manner by IgG antibody, we only presented the result of BRCA1 being pulled down by TRIM21 (Fig. 2F, Supplementary Fig. 2D). The ubiquitination level of BRCA1 was also elevated when exogenous TRIM21 was transfected (Fig. 2G). According to these findings, it was attempted to explore whether TRIM21 and BRCA1 could be colocalized in TNBC cells. Double immunofluorescent staining revealed the colocalization of TRIM21 and BRCA1 proteins in MDA-MB-231 cells. The spatial positions of BRCA1 (red) and TRIM21 (green) were stained with 4′,6-diamidino-2-phenylindole (DAPI) (blue) (Fig. 2H). The TRIM21 and BRCA1 proteins showed colocalized mainly in nuclear and exhibited a correlative intensity analyzed by ImageJ software with colocalization plugin JACoP (Fig. 2I). Taken together, these results demonstrated that TRIM21 mediated BRCA1 degradation by the ubiquitin-proteasome pathway and indicated that TRIM21 could play a negative role in TNBC cells, impairing the DNA repair capability by degrading BRCA1.

### TRIM21 affected the sensitivity of TNBC cells to Olaparib

**Fig. 3:**
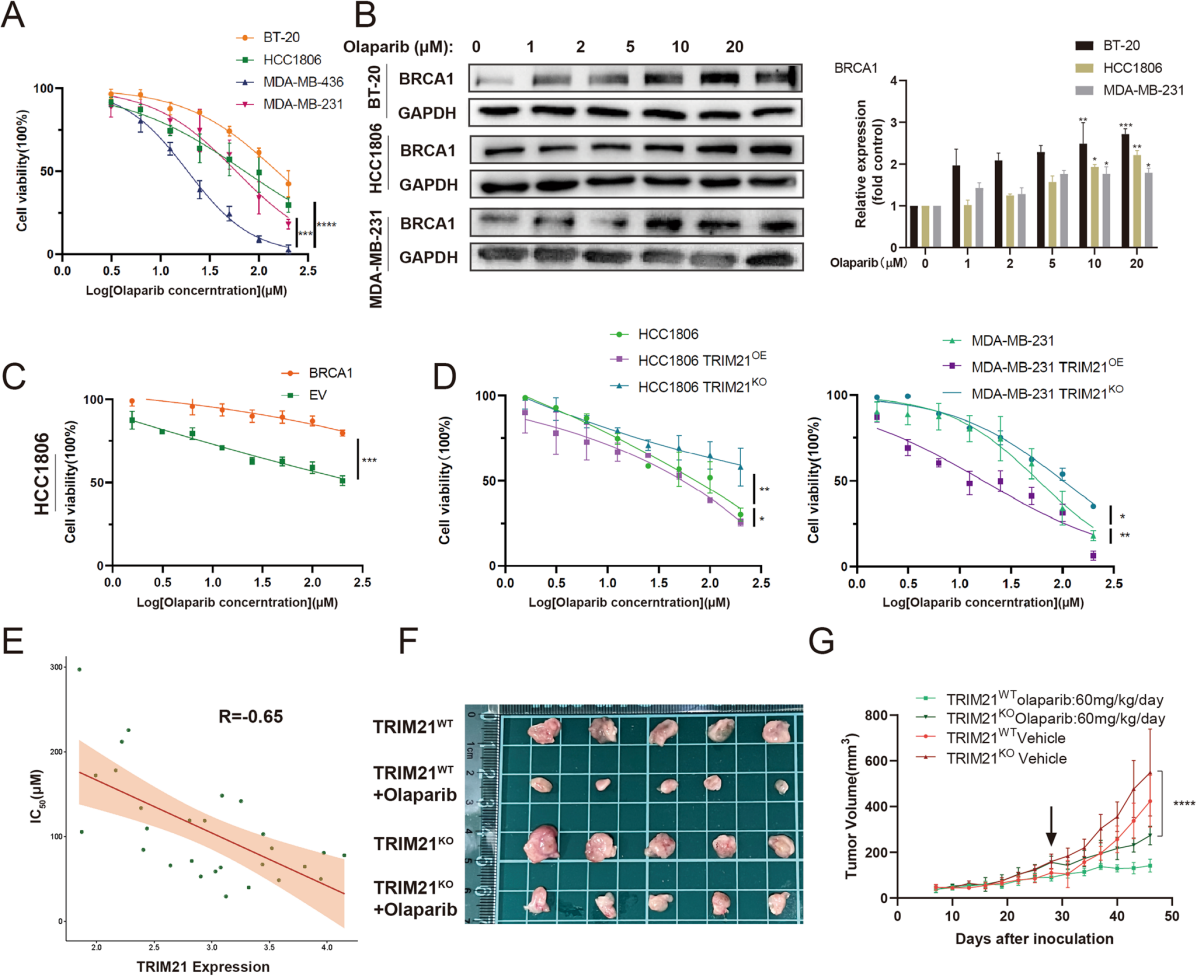
TRIM21 affects the sensitivity of TNBC to Olaparib. **A** The sensitivity of several TNBC cell lines was confirmed by the CCK-8 assay. **B** Treatment of Olaparib induced the upregulation of BRCA1. **C** The sensitivity of HCC1806 to Olaparib after transfecting exogenous BRCA1 was detected by CCK-8. **D** TRIM21^OE^ and TRIM21^KO^ TNBC cell lines possess different sensitivity toward Olaparib. **A**, **C**, and **D** Data are shown as the mean ± SD of at least three independent experiments. **E** Correlation analysis between the TRIM21 expression level and IC_50_ to Olaparib of various breast cancer cell lines. *P* = 0.0019. **F**, **G** MDA-MB-231 TRIM21^KO^ and TRIM21^WT^ cells (1 × 10^6^ cells/mouse) were injected into the right fourth breast pad. The mice were randomly divided into four groups with different setups. Tumor volumes were measured every 3 days. Data were shown as mean ± SD. The significant level was identified by *P < 0.05, **P < 0.01, ***P < 0.001 and ****P < 0.0001. Two-way ANOVA analysis is used for all cell viability curves (**A**, **C** and **D**) and tumor growth curves (**G**).

A previous study indicated that BRCA1-deficient breast cells exhibited increased sensitivity to Olaparib^24^. Moreover, Olaparib has been approved for the treatment of germline BRCA1/2 mutation breast cancer^25^. Our study confirmed this phenomenon in several TNBC cell lines through the cell counting kit-8 (CCK-8) assay. The BRCA1 mutated TNBC cell line MDA-MB-436 appeared remarkably sensitive to Olaparib. In contrast, other BRCA1-proficient cell lines were found relatively resistant to Olaparib (Fig. 3A). It was revealed that the BRCA1 expression level increased along with the elevation of Olaparib dose in these cell lines (Fig. 3B) which was also explicable that tumor cells strengthened the ability to repair DNA damage by upregulating BRCA1 when PARP was inhibited. Moreover, the enhanced capability in the HR repair pathway by transfecting exogenous BRCA1 affected the sensitivity of TNBC cells to Olaparib (Fig. 3C). To further explore whether TRIM21 expression level could influence the therapeutic efficacy of Olaparib, the half maximal inhibitory concentration (IC_50_) values of TRIM21^OE^ and TRIM21^KO^ TNBC cell lines to Olaparib were examined and listed in Supplementary Table 2. TRIM21^OE^ TNBC cells were found relatively sensitive to Olaparib compared with TRIM21^WT^ cells, while TRIM21^KO^ cells were relatively resistant (Fig. 3D). Correlation analysis showed a negative correlation between the cell sensitivity to Olaparib and TRIM21 expression level in breast cancer cell lines (R = −0.65). Data were collected from Cancer Cell Line Encyclopedia (CCLE) and Genomics of Drug Sensitivity (GDSC) databases (Fig. 3E). Further evidence was provided through in vivo experiments: TRIM21^KO^ MDA-MB-231 xenograft tumor exhibited enhanced proliferation compared with the parental cell line xenograft tumor. Olaparib treatment still reduces the tumor volume of the TRIM21^KO^ group in BALB/C nude mice (Fig. 3F, G). However, the anti-tumor rate in the TRIM21^WT^ group reached 67%, while it only reached 51.2% in the TRIM21^KO^ group. Collectively, TRIM21 mediated the sensitivity of TNBC cells to Olaparib, confirming its prognostic value. Moreover, the upregulation of TRIM21 could be advantageous for TNBC treatment.

### Sorafenib increased the sensitivity of TNBC cells to Olaparib through TRIM21

**Fig. 4:**
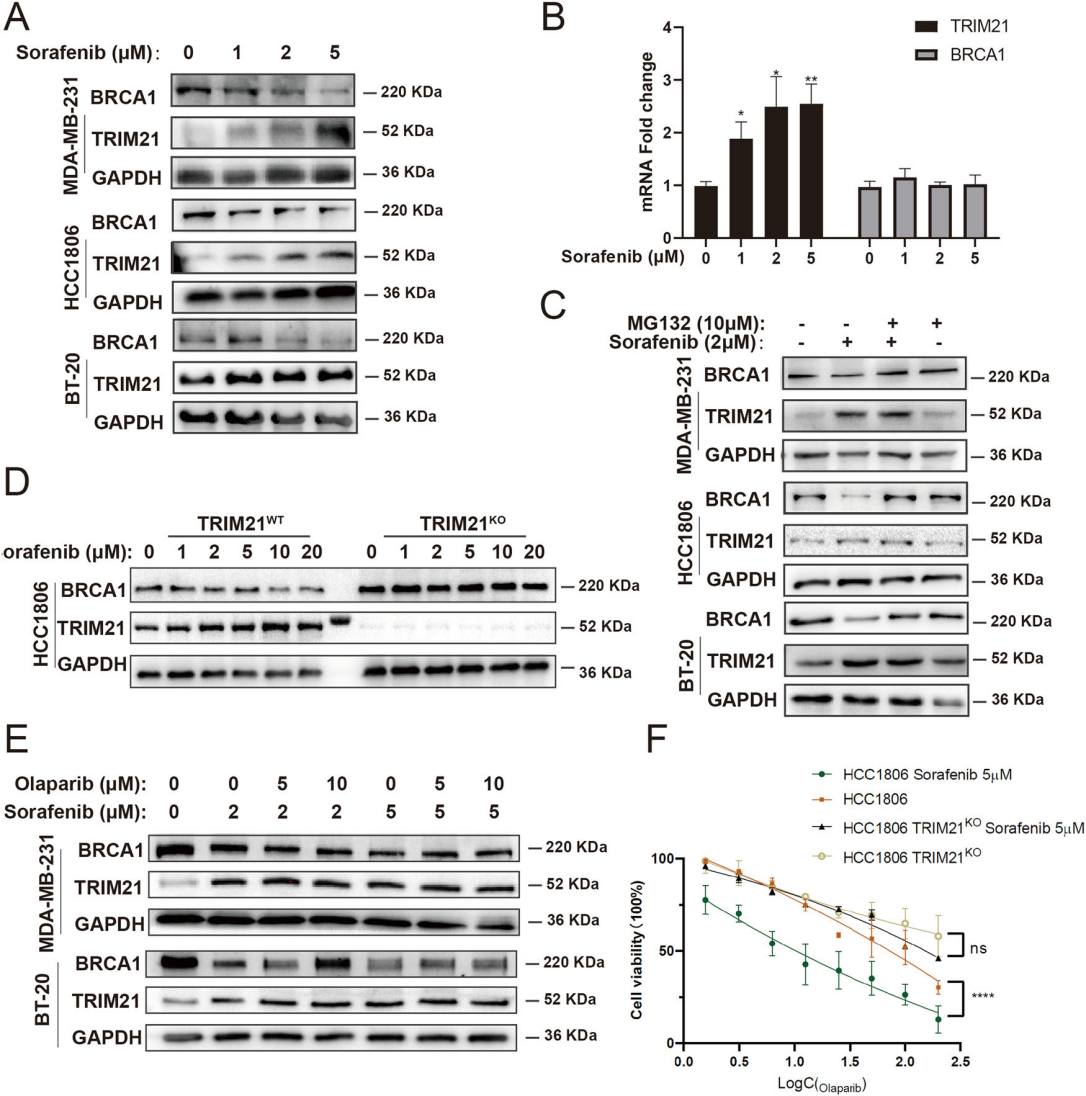
Sorafenib increases the sensitivity of TNBC cells to Olaparib through TRIM21. **A** Sorafenib treatment (24 h) increased the expression level of TRIM21 and decreased the protein level of BRCA1 in TNBC cells. **B** The mRNA expression level fold change of TRIM21 and BRCA1 compared to GAPDH of HCC1806. One-way ANOVA is used. **C** Pretreating MG132 for 2 h reversed the effect of Sorafenib in TNBC cells. **D** The expression level of BRCA1 in TRIM21^WT^ and TRIM21^KO^ TNBC cells treated with increasing concentration of Sorafenib for 24 h. **E** The expression level of BRCA1 with the combined treatment of Olaparib and Sorafenib. **F** The combined lethal effect of Sorafenib and Olaparib was subdued in the absence of TRIM21 measured by CCK-8. Data of (**B**) and (**F**) are shown as the mean ± SD of at least three independent experiments. The significant level was identified by ns: not significant; *P < 0.05, **P < 0.01, ***P < 0.001 and ****P < 0.0001. Two-way ANOVA analysis is used for cell viability curves.

Using small-molecule screening, it was found that Sorafenib, a standard therapy for hepatoma carcinoma, increased TRIM21 expression level. Simultaneously, the BRCA1 protein level was reduced (Fig. 4A). Moreover, the transcriptional level of TRIM21 was elevated after administration, while the BRCA1 mRNA level remained unchanged (Fig. 4B). Consequently, it was speculated that Sorafenib could regulate BRCA1 expression at the post-translation level probably through BRCA1-mediated ubiquitination degradation. Thus, TNBC cells were pretreated with MG132 as a proteasome inhibitor. The BRCA1 expression level remained unchanged after treatment with Sorafenib and MG132 (Fig. 4C). Moreover, Sorafenib failed to decrease the BRCA1 protein level in TRIM21^KO^ TNBC cells (Fig. 4D). As Olaparib treatment leads to the upregulation of BRCA1(Fig. 3B), so we wanted to explore whether Sorafenib treatment could reverse this phenomenon. Results revealed that Sorafenib downregulated BRCA1 protein expression even when combined with Olaparib (Fig. 4E). Furthermore, the combined lethal effect of Sorafenib and Olaparib on TNBC cells was subdued in the absence of TRIM21 (Fig. 4F). Taken together, these data revealed that Sorafenib increased the sensitivity of TNBC cells to Olaparib through TRIM21-mediated ubiquitination degradation of BRCA1 and suggested the potential of combined therapy.

### Olaparib and Sorafenib showed a synergic effect in vitro and in vivo

**Fig. 5:**
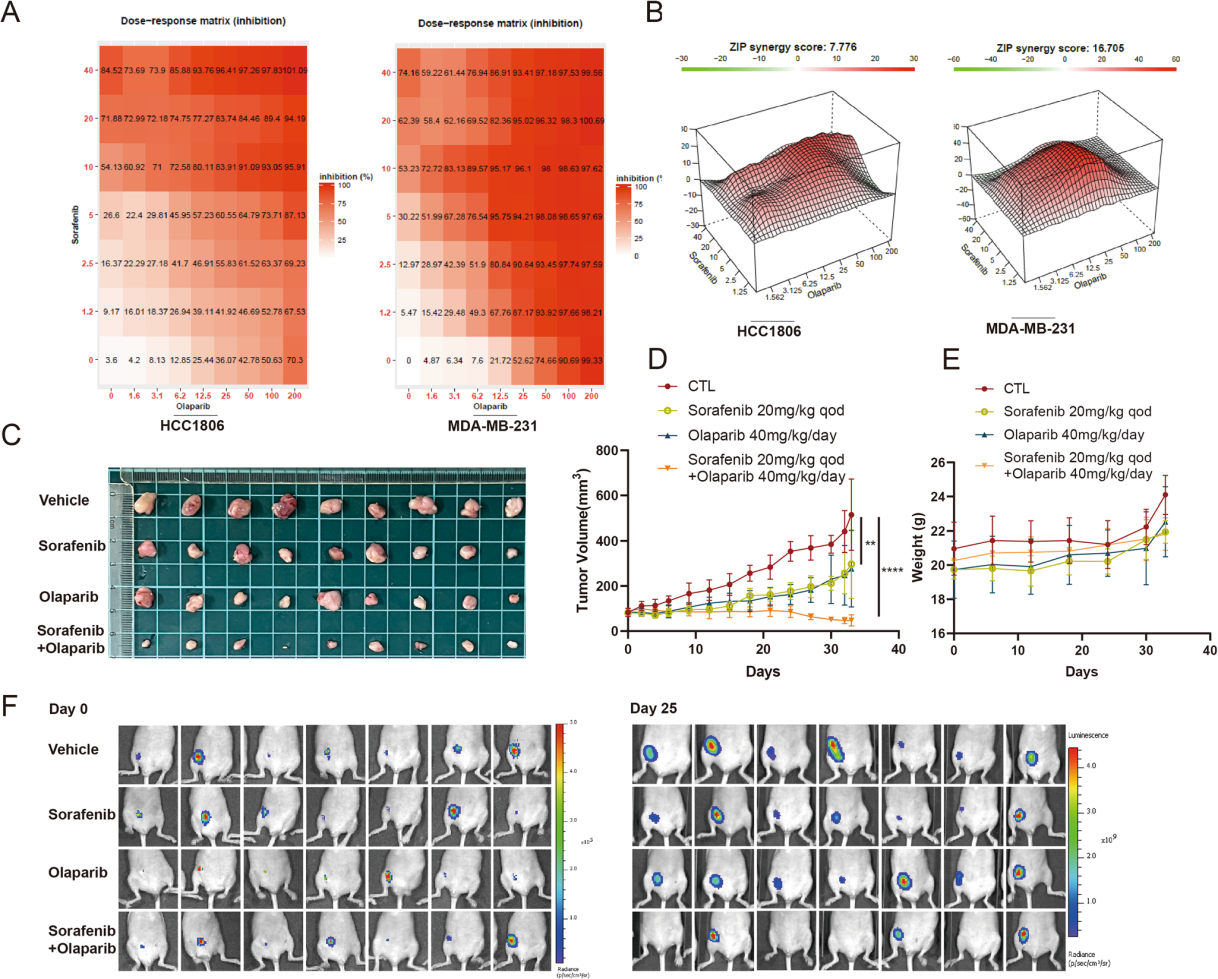
Olaparib and Sorafenib show a synergic effect in vitro and in vivo. **A** The dose-response matrixes by SynergyFinder are based on CCK-8 results of HCC1806 and MDA-MB-231. Data are shown as the mean value of at least three independent experiments. **B** The synergy score by the ZIP model provided by SynergyFinder is based on the matrixes. **C**–**F** MDA-MB-231-Luc (1 × 10^7^ cells/mouse) were injected into the right fourth breast pad. The mice were randomly divided into four groups with different setups. **C**, **D** Tumor volume. **E** Body weight. **F** In vivo bioluminescence. Data are shown as the mean ± SD. The significant level was identified by *P < 0.05, **P < 0.01, ***P < 0.001 and ****P < 0.0001. Two-way ANOVA analysis is used for tumor growth curves (**D**).

Finally, the combined efficacy of Sorafenib and Olaparib was confirmed in vitro and in vivo. The TNBC cell viability was evaluated using the CCK-8 assay. Both monotherapy and combined therapy were performed to obtain the inhibition rates at different concentrations. The results were presented as dose-response matrices by SynergyFinder. It showed that a higher dosage of Sorafenib caused a higher inhibition of cell viability in combination with Olaparib treatment (Fig. 5A). To further explore the synergistic effect of Sorafenib and Olaparib, based on the matrices, the synergy score was calculated by the ZIP model provided by SynergyFinder. The synergistic effects of all Sorafenib-Olaparib combinations were found, and more prominent synergistic effects were detected in the middle gradient concentrations (Fig. 5B). The synergistic effect was also confirmed in orthotopic MDA-MB-231-Luc-derived xenografts. The combination of Sorafenib and Olaparib significantly inhibited the tumor volume (Fig. 5C, D). The combination index (CI) < 1, calculated according to Loewe’s additivity principle, indicates the synergistic effect of Olaparib and Sorafenib in vivo. Ex vivo bioluminescence images further displayed strong Luc signals in vehicle-treated mice and diminished or even disappeared signals in mice treated with combined therapy (Fig. 5F). Moreover, there was no significant difference in body weight between the combined therapy group and the monotherapy group, indicating low toxicity of combined therapy (Fig. 5E). Collectively, these results demonstrated the synergistic effect of Olaparib and Sorafenib in treating TNBC and revealed the application prospects of this combined therapy.

### The combination of Olaparib and Sorafenib resulted in DNA damage, cell cycle arrest, and apoptosis

**Fig. 6:**
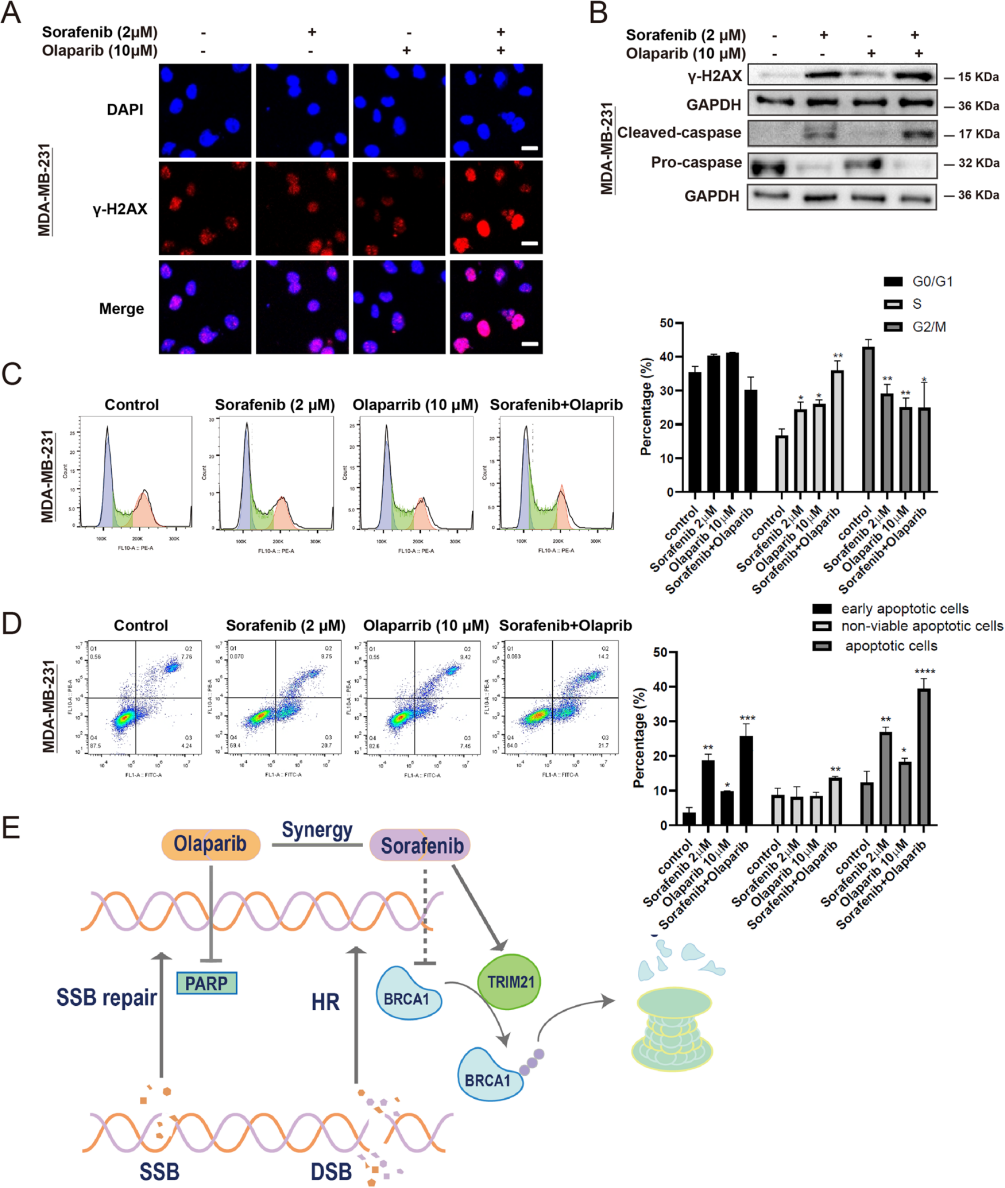
The combination of Olaparib and Sorefenib results in DNA damage, cell cycle arrest, and apoptosis. **A** The level of DNA double-strand breaks was indicated by γ-H2AX foci by immunofluorescence. Bar length: 10 μm. **B** The protein expression level of cleaved caspase and γ-H2AX. **C** Cell cycle rate under different drugs obtained through flow cytometry. **D** The cell apoptosis rate was measured by flow cytometry. The gating strategy is provided in Supplementary Fig. 2. **C**, **D** Data are shown as the mean ± SD. One-way ANOVA analysis is used. **E** The mechanism of the synergic effect between Olaparib and Sorafenib. Sorafenib indirectly inhibits BRCA1 through TRIM21-mediated ubiquitination, thus inactivating the homologous recombination (HR) repair pathway. Meanwhile, Olaparib inhibits PARP, which is mainly responsible for SSBs repair. The combination of these two drugs is synergistically lethal to TNBC cells. The significant level was identified by *P < 0.05, **P < 0.01, ***P < 0.001 and ****P < 0.0001.

To integrally confirm that the cytotoxic effects of combined therapy could be correlated with DNA damage, which was analyzed by evaluating the formation of γ-H2AX foci (Fig. 6A). The combination of Sorafenib and Olaparib induced the stronger staining of γ-H2AX foci, indicating a higher level of DSBs. Importantly, the monotherapy of Olaparib only led to slight DSBs, which could be a result of the upregulation of BRCA1. Moreover, the γ-H2AX expression level was further analyzed by Western blotting, in which the results were consistent with those of fluorescence assay (Fig. 6B). Cell cycle arrest is mainly induced to facilitate DNA repair, and the unrecoverable DNA damage triggers apoptosis. Therefore, the effects of combined therapy on cell cycle arrest and apoptosis were also explored by Western blotting and flow cytometry. The combined therapy increased the percentage of TNBC cells at the S phase and decreased the proportion of TNBC cells at the G2/M phase compared with the control group (Fig. 6C). The monotherapy using Sorafenib increased the early apoptosis rate. The monotherapy using Olaparib slightly increased the early apoptosis rate. In contrast, the combined therapy significantly increased the early and late non-viable apoptosis rates of TNBC cells (Fig. 6D). The gating/sorting strategies are provided in the Supplementary Fig. 2. The cleaved caspase expression level was significantly upregulated, as confirmed by Western blotting (Fig. 6B). Collectively, the combined therapy could lead to enhanced DNA damage, cell cycle arrest, and apoptosis of TNBC cells.

## Discussion

It is widely accepted that a high rate of metastasis, poor prognosis, high heterogeneity, and lack of therapeutic targets are the main clinical and pathological characteristics of TNBC. Some scholars attempted to eliminate the difficulties, such as “resistance” and “tumor heterogeneity” in cancer therapy. Thus, the concept of synthetic lethality has been put forward and has highlighted the clinical application of PARP inhibitors. Our research demonstrated that the combination of Sorafenib and Olaparib exhibited a synthetic lethality effect. Sorafenib could inhibit BRCA1 by TRIM21-mediated ubiquitination degradation, leading to defective HR. Consequently, the DNA lesions caused by Olaparib could not be repaired, resulting in cell cytotoxicity, which could eventually lead to the apoptosis of TNBC cells (Fig. 6E). Another study also verified that in hepatocellular carcinoma, Olaparib broadly suppressed the DNA damage repair and global pluripotent transcriptional network, thereby reinforcing Sorafenib^26^. Collectively, Olaparib and Sorafenib may be synergistic in a variety of ways.

For the future treatment of breast cancer, clinical advances in PARP inhibitors and immunotherapy in TNBC have brought hope to TNBC patients. They have shown significant improvements in progression-free survival and overall survival, both as single agents and in combination with chemotherapy. However, the results of a phase III clinical trial in 2014 showed that Olaparib in combination with chemotherapy did not benefit patients^27^. It is suggested that the application of PARP inhibitors with PD-L1 inhibitors requires rigorous biomarker guidance, including BRCA1/2 mutations, PD-L1 expression levels in tumor tissues, tumor genomic microsatellite stability (MSS/MSI), and mutational load (TMB) assessment. However, the genomic landscape of TNBC is heterogeneous and complex^28^. Thus, it is almost impossible to expect a single drug regimen to control the malignant growth of TNBC. Therefore, starting from the molecular marker mapping of TNBC, further molecular subtyping of TNBC is critical to guide guiding individualized patient treatment.

A growing body of evidence showed that TRIM21 and its dysregulation could be involved in the initiation and progression of multiple types of human cancer, and TRIM21 could serve as either an oncogene or tumor suppressor, varying according to the carcinogenesis effectors and the types of cancer^29^. The present study also confirmed the role of TRIM21 as a tumor suppressor in TNBC and its key role in mediating the combined therapeutic effect of Sorafenib and Olaparib. Besides, there is a pronounced significance, demonstrating that TRIM21 directly mediates ubiquitinated degradation of BRCA1 that is currently “undruggable”. Thus, detailed interactions, such as binding sites between TRIM21 and BRCA1, are needed. Recently, a “Trim-Away” technique, which is applicable to rapidly and selectively degrade endogenous proteins, harnessed TRIM21 to drive this process by introducing antibodies against proteins of interest and exogenous TRIM21^30^. Hence, modulating the degradation process of BRCA1, as well as other vital proteins in cell activity by TRIM21, may be feasible for cancer treatment and needs to be more profoundly investigated.

Nevertheless, there are still some mechanisms in the present study that need to be clarified. The mechanisms of Sorafenib in modulating TRIM21 have remained elusive. It could be related to the transcriptional regulation, while the transcription factors were elusive. Bioinformatic evidence showed that the BARD1 may have interaction with TRIM21. Additional studies will be required to confirm the hypothesis and elucidate the mechanisms. Although our study has demonstrated the significant role of TRIM21 in drug-combined therapy and in mediating BRCA1 ubiquitination degradation, the extensive downstream substrates have not been assessed and no comparative genomics analysis was conducted. The tumor suppressor role and inner mechanisms of TRIM21 in TNBC or other types of breast cancer should be further verified and elaborated by further experimental evidence and omics analysis. Besides, the combined efficacy of Olaparib and Sorafenib should be verified in more patient-derived xenograft cancer models.

In conclusion, the findings of the present study expanded the possibility of the combined treatment of Olaparib and Sorafenib for TNBC, especially for BRCA1-proficient TNBC patients, and facilitated the exploration of one possible inner mechanism. Moreover, it was revealed that TRIM21 is not only a target protein and possesses great potential as a therapeutic target for diverse types of cancer, but also may be a tool enzyme with promise from bench to bedside.

## Methods

### Cell culture

All TNBC cell lines were provided by PharmaLegacy Laboratories (Shanghai, China). All these cell lines have been authenticated within the last 3 years by SNP profiling and all experiments were performed with mycoplasma-free cells. The cultural conditions are listed below. MDA-MB-231 (RRID: CVCL_0062): Leibovitz’s L-15 medium (Corning, US) containing 10% heat-inactivated fetal bovine serum (FBS, Gibco, US) and 1% penicillin-streptomycin (PS, Gibco), at 37 °C in a humidified incubator containing 100% air. MDA-MB-436 (RRID: CVCL_0623): L-15 medium containing 10% FBS and 1% PS and 10 μg/mL insulin at 37 °C, 100% air. HCC1806 (RRID: CVCL_1258): RPMI-1640 medium containing 5% FBS and 1% PS at 37 °C, 95% air, and 5% CO_2_. BT-20 (RRID: CVCL_0178): MEM medium containing 10% FBS and 1% PS at 37 °C, 95% air, and 5% CO_2_. HEK 293T (RRID: CVCL_LF52): DMEM medium containing 10% FBS and 1% PS at 37 °C, 95% air, and 5% CO_2_. Phosphate-buffered saline (PBS) and trypsin-EDTA solution were obtained from Corning.

### Cell proliferation and colony formation assays

The relative proliferation rates of TNBC cells were measured by the Cell Titer-Lumi™ Luminescent Cell Viability Assay kit (CTG, Beyotime, China). For colony formation Assays, TNBC cells were seeded in a 6-well plate at the concentration of 2000 cells per well. On day 10, colonies were fixed with 4% formaldehyde for 10 min and then stained with crystal violet for 10 min.

### Cell migration and invasion assays

To test the migration ability, TNBC cells were seeded at 5 × 10^5^ cells per well in a 6-well plate. On day 2, wound healing was performed by creating a physical gap within a cell monolayer with tips and replacing the medium with a 2% FBS medium. Live cell images were then taken at 0 and 24 h, and the gap closure rate was analyzed using ImageJ. To obtain the invasion abilities, 100 μL of 1:8-diluted Matrigel (Corning) was coated onto chambers. A total of 1 × 10^5^ TNBC cells in 100 μL FBS-free medium were seeded in the upper chamber (Corning, 354234). The bottom lower chamber was filled with 600 μL medium containing 20% FBS. After incubation of the cells at 37 °C for 36 h, the bottom surface of the chamber was fixed with 4% formaldehyde for 10 min and then stained with DAPI for 5 min. Results were obtained by fluorescence imaging using an Olympus BX41 microscope.

### Transient transfection and lentiviral infection

Transient transfections and lentiviral infection, including sgRNA knockout, were performed according to the protocol of Lipofectamine 3000 (Thermo Fisher Scientific, US). The information of plasmids is listed: h-TRIM21 (pCDH-CMV-MCS-EF1-copGFP); h-TRIM21 (pCMV3-ORF-Myc); h-BRCA1 (pDEST-Flag); h-TRIM21 (Plenticrispr v2); h-TRIM21-ligase dead (pLNCX2 ER:ras). The detailed plasmid information is provided in Supplementary Table 1.

### Cell viability assays

All experiments involved in cell viability assays were conducted according to the following protocols of Cell Counting Kit-8 (CCK-8, Beyotime). The TNBC cells at a concentration of 5000 cells per well were seeded in a 96-well plate. Then, 24 h later, a series concentration of chemical reagents was added by 2-fold gradient dilution. The absorbances of cells (450 nm) after 72 h were examined with a microplate reader according to the instructions.

### In vivo experiments

Female BALB/C nude mice, which were aged 6–8 weeks and weighed 18–22 g, were obtained from Shanghai Lingchang Biotechnology Co., Ltd. (Shanghai, China), and they were housed with adequate food and water under specific pathogen-free (SPF) conditions and kept at 22–25 °C for 5 days acclimation period under 12–12 h light-dark cycles. For the combination effect experiment of Sorafenib (PubChem CID: 216239) and Olaparib (PubChem CID: 23725625), the TNBC cell line MDA-MB-231-GFP-Luc used in this experiment was cultured in an L-15 medium with 10% fetal bovine serum (FBS) in an incubator without CO_2_ at 37 °C. Before continuous cell culture for 10 passages, about 1 × 10^7^ MDA-MB-231-GFP-Luc cells were suspended in 100 µL phosphate-buffered saline (PBS), mixed with 100 µL Matrigel (Cat. No. 354234; Corning Inc., Corning, NY, USA), and seeded into the right fourth breast pad of BALB/C nude mice. Mice were anesthetized with 2–5% isoflurane before inoculation. All mice with a tumor volume of within 80 mm^3^ were randomly divided into four groups. Olaparib (40 mg/kg) was administered per day. Sorafenib (20 mg/kg) was administered every other day. At 0 and 25 days after implantation, bioluminescence imaging (IVIS spectrum in vivo imaging system; PerkinElmer, Waltham, MA, USA) was used to detect tumor growth. Tumor volume was measured by a vernier caliper and calculated every 3 days using the following formula: V = 1/2LW^2^, where V, L, and W indicate the volume, length, and width of the tumor, respectively. To compare the sensitivity of TRIM21^KO^ and TRIM21^WT^ MDA-MB-231 cell lines to Olaparib, in vivo studies were conducted following the similar procedures mentioned above. Only the number of inoculated cells was 1 × 10^6^; Olaparib (60 mg/kg) was administered per day. The experiment ended on Day 25 after the first drug exposure. Mice were anaesthetized and tumor samples were collected in formaldehyde solution. All animal experiments were approved and conducted by the Guide of the Institutional Animal Care and Use Committee of PharmaLegacy Laboratories Co., Ltd. with accreditation of the Association for Assessment and Accreditation of Laboratory Animal Care (AAALAC).

### Western blot

Total cell lysates were prepared in ice-cold RIPA buffer (Thermo) supplemented with 1% protease inhibitor (P1006, Beyotime), and then added 10% SDS. The protein extracts were separated by SDS-PAGE and were then transferred to polyvinylidene difluoride (PVDF) membranes, followed by blocking with 5% skimmed milk in TBST buffer (Tris-buffered saline and 0.1% Tween 20) for 2 h at room temperature. Incubating the primary antibody overnight at 4 °C. The following day, membranes were incubated with anti-mouse or anti-rabbit peroxidase-conjugated secondary antibodies for 2 h at room temperature. Image Lab (Bio-Rad Laboratories, Hercules, CA, USA) was employed for the final image processing. All antibodies used are listed: Anti-TRIM21 (1:1000, ab207728, Abcam); Anti-TRIM21 (1:1000, 12108-1-AP, Proteintech); Anti-BRCA1 (1:200, D-9, Santa Cruz); Anti-GAPDH (1:2000, 10494-1-AP, Proteintech); Anti-Caspase (1:1000, 9662S, Cell Signaling Technology); Anti-Cleaved caspase (1:1000, 9661S, Cell Signaling Technology); Anti-γH2AX (1:1000, 9718T, Cell Signaling Technology) Anti-Flag (1:1000, 66008-3-Ig, Proteintech); Anti-ubiquitin (1:1000, 10201-2-AP, Proteintech); HRP-conjugated anti-rabbit (1:10000, A0208, Beyotime); HRP-conjugated anti-mouse (1:1000, A0216, Beyotime); Alexa Fluor 555 anti-mouse (1:1000, A0473, Beyotime); Alexa Fluor 488 anti-rabbit (1:1000, AO423, Beyotime); Alexa Fluor 555 anti-rabbit (1:1000, AO453, Beyotime). The uncropped scans are also uploaded in the Supplementary material.

### Quantitative RT-PCR

Total RNA from cells was extracted by using Trizol reagent (15596; Invitrogen, US) according to the instructions. RNA was then reverse transcribed to cDNA by using the PrimeScript™ RT Master Mix (TaKaRa, Japan). Complementary gene expression was determined by SYBR-Green PCR assay (TaKaRa) in a CFXTM Real-Time Thermal cycler (Bio-Rad, Hercules, US). Data were analyzed with the 2^−^^ΔΔCt^ method, normalized to GAPDH. The sequences of the primer were listed: h-Trim21 (F: 5’-TCAGCAGCACGCTTGACAAT-3’, R: 5’-GGCCACACTCGATGCTCAC-3’); h-BRCA1 (F:5’-AAGGTTGTTGATGTGGAGGAG-3’, R:5’-CAGAGGTTGAAGATGGTATGTTG-3’); h-GAPDH (F:5’-GGGGAGCCAAAAGGGTCATCATCT-3’, R5’-GAGGGGCCATCCACAGTCTTCT-3’).

### Co-immunoprecipitation (CO-IP)

Co-immunoprecipitation experiment was performed according to the following instructions. First, the antibodies were immobilized to protein A/G agarose beads (Beyotime) for 2 h. Then, wash the beads with TBS buffer three times. Then cell lysates were prepared by RIPA and centrifuged at 13,000 × *g* for 5 min at 4 °C. The supernatants were transferred to new tubes and incubated with suitable antibodies-immobilized beads overnight at 4 °C with constant agitation. The immunoprecipitation was added to the SDS loading Buffer and further detected by western blot.

### Immunofluorescence and immunofluorescence colocalization

For Immunofluorescence, TNBC cells were seeded in the confocal dish at a density of 5 × 10^5^ cells per well. After 24 h, chemical regiments are added. The next day, cells were washed and fixed with 4% formaldehyde for 30 min at RT. Then cells were permeabilized with 1% Triton X-100 for 20 min. The non-specific binding sites were later blocked by blocking solution (Beyotime) for 2 h. The cells were incubated with specific primary antibodies overnight at 4 °C. After washing with PBS, the corresponding secondary antibodies conjugated to Alexa Fluor 555 and Alexa Fluor 488 were applied at room temperature for 1 h and then stained with DAPI (1:1000, Beyotime) for 5 min. The specimens were detected and imaged by a laser-scanning confocal microscope (Olympus, Japan).

### Flow cytometry

To test the cell cycle and apoptosis of TNBC, control (untreated) and treated cells were harvested from 6-well plates after 24 h of exposure to drugs. Harvested cells were washed twice with PBS and fixed with 70% ethanol on ice for 2 h. After cell fixation, cells were then washed and resuspended in 0.5 mL PBS containing propidium iodide (50 µg/mL) and DNase-free RNase (100 µg/mL) for 10 min. The cell cycle distribution was assessed by flow cytometry (CytoFlex S, Beckman, US). All data analyses were performed on Flowjo software to calculate the percentages of cells in the G0/G1, S, and G2/M phases of the TNBC cell cycle. Apoptosis was detected using the Annexin V-FITC apoptosis kit (C1062S, Beyotime) according to the instructions of the manufacturers.

### Bioinformatic analysis

Kaplan–Meier analysis: Kaplan–Meier Plotter is a comprehensive online tool that can be used to analyze the effects of 54,675 genes on survival in 21 cancer types (https://kmplot.com/analysis/). The integrated dataset consists of GSE11121, GSE12093, GSE12276, GSE1456, GSE16391, GSE16446, GSE16716, GSE17705, GSE17907, GSE 18728, GSE19615. We used it to obtain the relationship between TRIM21 and relapse-free survival (RFS) by breast cancer gene-chip data. Hazard ratios (HRs) and log-rank *P*-values were calculated. The prognostic value was considered statistically significant when the *P*-value was less than 0.05.

Pan-cancer analysis: correlation between TRIM21 and signaling pathways by Gene Set Cancer Analysis. Normalize the gene expression data obtained from The Cancer Genome Atlas (TCGA) to correct for variations in sequencing depth. Kyoto Encyclopedia of Genes and Genomes (KEGG) contains signaling pathways of interest. Using the Enrichr to perform the gene set enrichment analysis and perform statistical tests. Visualizing the differential expression of TRIM21 and the signaling pathway genes across different cancer types by heatmaps.

UbiBrowser prediction: Ubibrowser is an integrated database for predicting proteome-wide E3-substrate interactions. We use this database to predict the potential E3 ligases of BRCA1 and substrates of TRIM21 and visualize the score by heatmap.

TRIM21-BRCA1 Interaction analysis: The TRIM21-BRCA1 interaction analysis was conducted using the GeneMANIA database. GeneMANIA is a powerful online tool that integrates various functional genomics data to predict the functional relationships between genes and proteins based on computational algorithms and experimental data.

Correlation analysis: to analyze the correlation between the expression level of the TRIM21 gene and Olaparib sensitivity in breast cancer cells. TRIM21 gene expression in Breast cells was obtained from the Cancer Cell Line Encyclopedia (CCLE) database. The Olaparib sensitivity of different cancer cells was obtained from the Genomics of Drug Sensitivity (GDSC) database.

### Statistical analysis

All the experimental data were calculated and presented as the mean ± standard deviation of at least three independent experiments. Statistical analyses were performed using GraphPad 8.0 statistical software (La Jolla, CA, US). Experimental data obtained were analyzed using a one-way or two-way ANOVA of variance followed by Tukey’s multiple-range tests for significance. A statistically significant difference was considered under the situation of *P* < 0.05.

### Reporting summary

Further information on research design is available in the Nature Research Reporting Summary linked to this article.

### Supplementary information

Supplementary files

Reporting Summary

## Data Availability

Data will be made available upon reasonable request to xinhong@fudan.edu.cn.

## References

[1] Siegel RL, Miller KD, Fuchs HE, Jemal A (2022). Cancer statistics, 2022. CA Cancer J. Clin..

[2] Curtis C (2012). The genomic and transcriptomic architecture of 2,000 breast tumours reveals novel subgroups. Nature.

[3] Perou CM (2000). Molecular portraits of human breast tumours. Nature.

[4] Bertucci F (2008). How basal are triple-negative breast cancers?. Int. J. Cancer.

[5] Waks AG, Winer EP (2019). Breast cancer treatment: a review. JAMA.

[6] Hernandez-Aya LF (2011). Nodal status and clinical outcomes in a large cohort of patients with triple-negative breast cancer. J. Clin. Oncol..

[7] Robson M (2017). Olaparib for metastatic breast cancer in patients with a germline BRCA mutation. N. Engl. J. Med..

[8] Dobzhansky T (1946). Genetics of natural populations; recombination and variability in populations of *Drosophila pseudoobscura*. Genetics.

[9] Takaoka M, Miki Y (2018). BRCA1 gene: function and deficiency. Int. J. Clin. Oncol..

[10] Farmer H (2005). Targeting the DNA repair defect in BRCA mutant cells as a therapeutic strategy. Nature.

[11] Bryant HE (2005). Specific killing of BRCA2-deficient tumours with inhibitors of poly(ADP-ribose) polymerase. Nature.

[12] Shi Y, Jin J, Ji W, Guan X (2018). Therapeutic landscape in mutational triple negative breast cancer. Mol. Cancer.

[13] Brianese RC (2018). BRCA1 deficiency is a recurrent event in early-onset triple-negative breast cancer: a comprehensive analysis of germline mutations and somatic promoter methylation. Breast Cancer Res. Treat.

[14] Gonzalez-Angulo AM (2011). Incidence and outcome of BRCA mutations in unselected patients with triple receptor-negative breast cancer. Clin. Cancer Res..

[15] Huang N (2022). TRIM family contribute to tumorigenesis, cancer development, and drug resistance. Exp. Hematol. Oncol..

[16] Hillen, M. R. et al. Autoantigen *TRIM21*/Ro52 is expressed on the surface of antigen-presenting cells and its enhanced expression in Sjogren’s syndrome is associated with B cell hyperactivity and type I interferon activity. *RMD Open* **6**, e001184 (2020). 10.1136/rmdopen-2020-001184 PMC7431915 32540951

[17] Brauner S, Ivanchenko M, Thorlacius GE, Ambrosi A, Wahren-Herlenius M (2018). The Sjögren’s syndrome-associated autoantigen Ro52/TRIM21 modulates follicular B cell homeostasis and immunoglobulin production. Clin. Exp. Immunol..

[18] McEwan WA (2017). Cytosolic Fc receptor TRIM21 inhibits seeded tau aggregation. Proc. Natl Acad. Sci. USA.

[19] Alomari MJPR (2021). TRIM21—a potential novel therapeutic target in cancer. Pharmacol. Res..

[20] Su X (2021). The noncoding RNAs SNORD50A and SNORD50B-mediated TRIM21-GMPS interaction promotes the growth of p53 wild-type breast cancers by degrading p53. Cell Death Differ..

[21] Si W, Zhou J, Zhao Y, Zheng J, Cui L (2020). SET7/9 promotes multiple malignant processes in breast cancer development via RUNX2 activation and is negatively regulated by TRIM21. Cell Death Dis..

[22] Zhou W (2018). Decreased expression of TRIM21 indicates unfavorable outcome and promotes cell growth in breast cancer. Cancer Manag. Res..

[23] Baumann P, West SC (1998). Role of the human RAD51 protein in homologous recombination and double-stranded-break repair. Trends Biochem. Sci..

[24] Lee JM, Ledermann JA, Kohn EC (2014). PARP inhibitors for BRCA1/2 mutation-associated and BRCA-like malignancies. Ann. Oncol..

[25] Tutt ANJ (2021). Adjuvant Olaparib for patients with BRCA1- or BRCA2-mutated breast cancer. N. Engl. J. Med..

[26] Yang XD (2021). PARP inhibitor Olaparib overcomes Sorafenib resistance through reshaping the pluripotent transcriptome in hepatocellular carcinoma. Mol. Cancer.

[27] Xie Y (2021). Public health insurance and cancer-specific mortality risk among patients with breast cancer: a prospective cohort study in China. Int. J. Cancer.

[28] Bianchini G, De Angelis C, Licata L, Gianni L (2022). Treatment landscape of triple-negative breast cancer—expanded options, evolving needs. Nat. Rev. Clin. Oncol..

[29] Alomari M (2021). TRIM21—a potential novel therapeutic target in cancer. Pharmacol. Res..

[30] Clift D (2017). A method for the acute and rapid degradation of endogenous proteins. Cell.

