# Supplementary material for: TRIM21 mediates the synergistic effect of Olaparib and Sorafenib by degrading BRCA1 through ubiquitination in TNBC
Source: NPJ Breast Cancer. 2023 Oct 20;9:85. doi: 10.1038/s41523-023-00588-1 (PMC10589312; doi:10.1038/s41523-023-00588-1)
Supplement: Supplementary file 1 — Supplementary files [file 41523_2023_588_MOESM1_ESM.pdf]

Supplementary Table S1: plasmids information

Gene Name	Vector	Type
h-TRIM21	pCDH-CMV-MCS-EF1-copGFP	Overexpression Plasmid (for lentivirus infection)
h-TRIM21	pCMV3-ORF-Myc	Overexpression Plasmid
h-BRCA1	pDEST-Flag	Overexpression Plasmid
h-TRIM21	Plenticrispr v2	SgRNA Plasmid
h-TRIM21-ligase dead	pLNCX2 ER:ras	Overexpression Plasmid

Supplementary Table S2: IC_{50} of TNBC cell lines

Parental Cell lines	Gene expression/mutation	IC_{50} (μM)
MDA-MB-231	TRIM21 WT	68.78
MDA-MB-231	TRIM21 OE	31.88
MDA-MB-231	TRIM21 KO	142.31
HCC1806	TRIM21 WT	71.87
HCC1806	TRIM21 OE	45.67
HCC1806	TRIM21 KO	169.20
BT-20	TRIM21 WT	153.1
MDA-MB-436	BRCA1 mutation	18.86

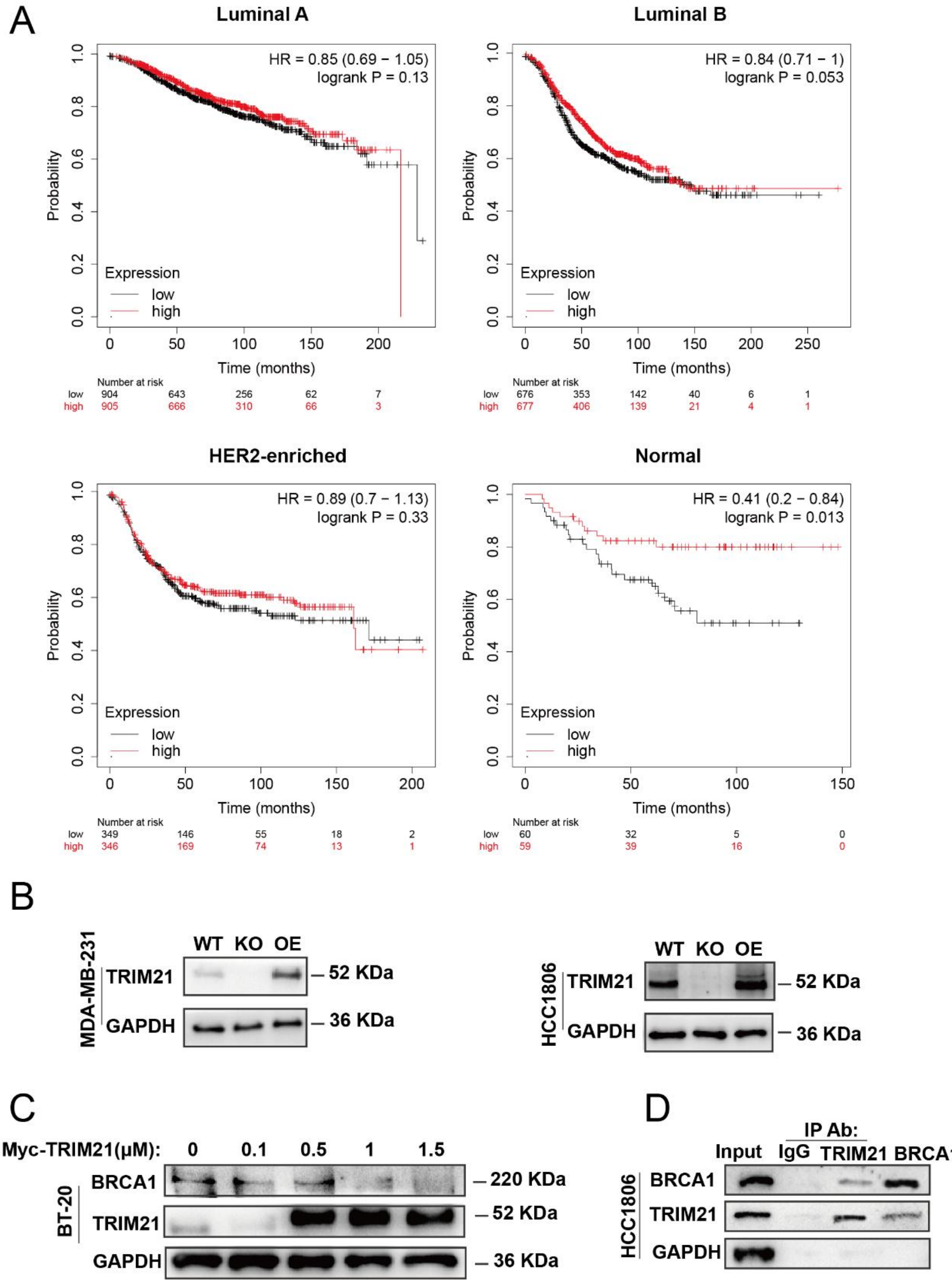

Supplementary Figure 1. (A) The correlation between the expression level of TRIM21 and relapse-free survival of Luminal A, Luminal B, HER2-enriched, and normal breast cancer types analyzed by gene chip. (B) WB verification for TRIM21^{KO} and TRIM21^{OE} TNBC cells. (C). The endogenous BRCA1 protein level was examined after transfecting with exogenous TRIM21.

(D). The CO-IP result between TRIM21，BRCA1 and GAPDH were confirmed by WB.

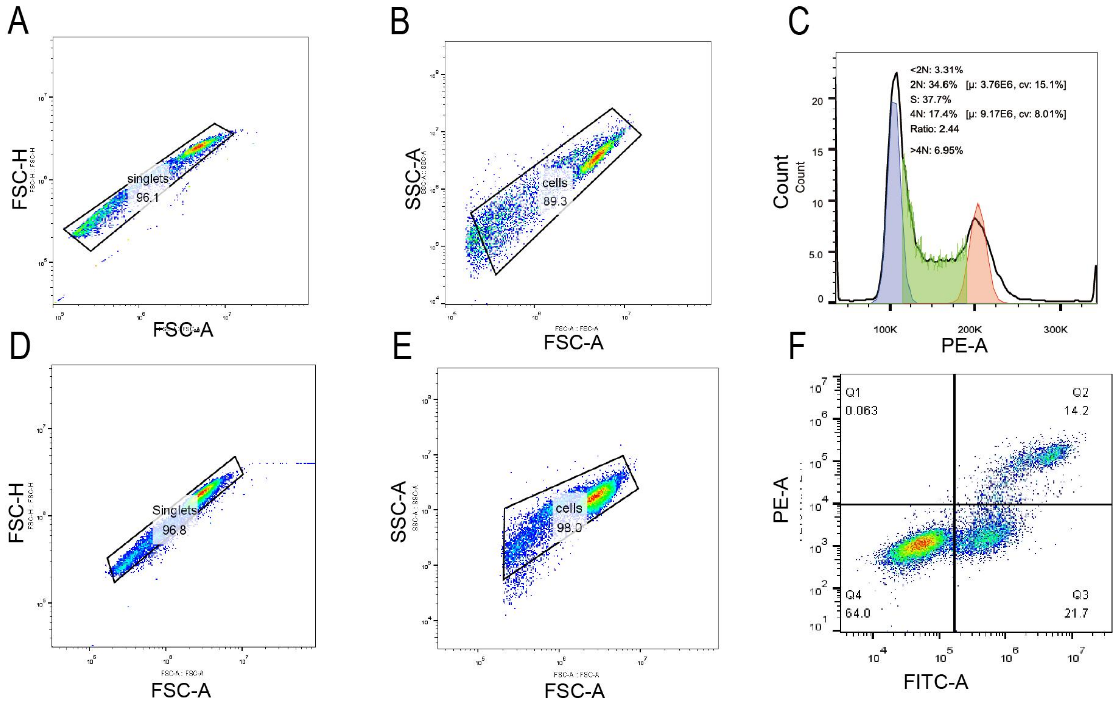

Supplementary Figure 2. Gating strategies used in flow cytometric analysis of tumor cell lines. A and D:Debris were gated out. B and E: non-viable cells were gated out. C:cell cycle are divded by the Watson Pragmatic algorithm provided by FlowJo v10. Apoptosis are gated by PE-A and FITC-A. Q2: non-viable apoptotic cells. Q3: early apoptotic cells. Q4: non-apoptotic cells.

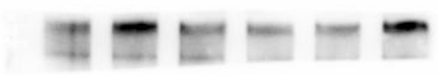
Marker 170kDa-
-220kDa
Figure 2C BRCA1

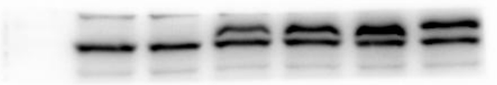
Marker 55kDa-
-52kDa
Figure 2C TRIM21

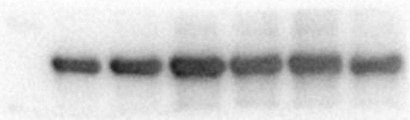
Marker 40kDa-
-36kDa
Figure 2D GAPDH

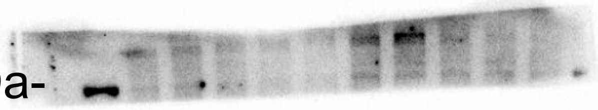
Marker 170kDa-
-220kDa
Figure 2E BRCA1

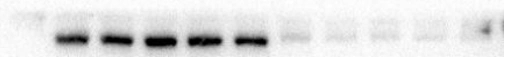
Marker 55kDa-
-52kDa
Figure 2E TRIM21

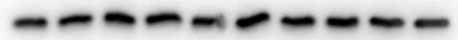
Marker 40kDa-
-36kDa
Figure 2E GAPDH

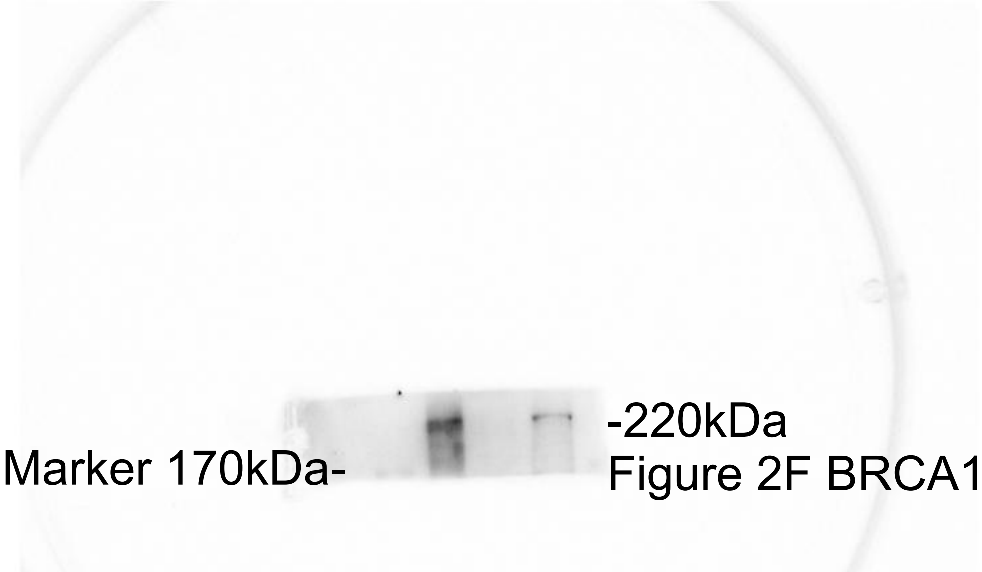
Marker 170kDa-
-220kDa
Figure 2F BRCA1

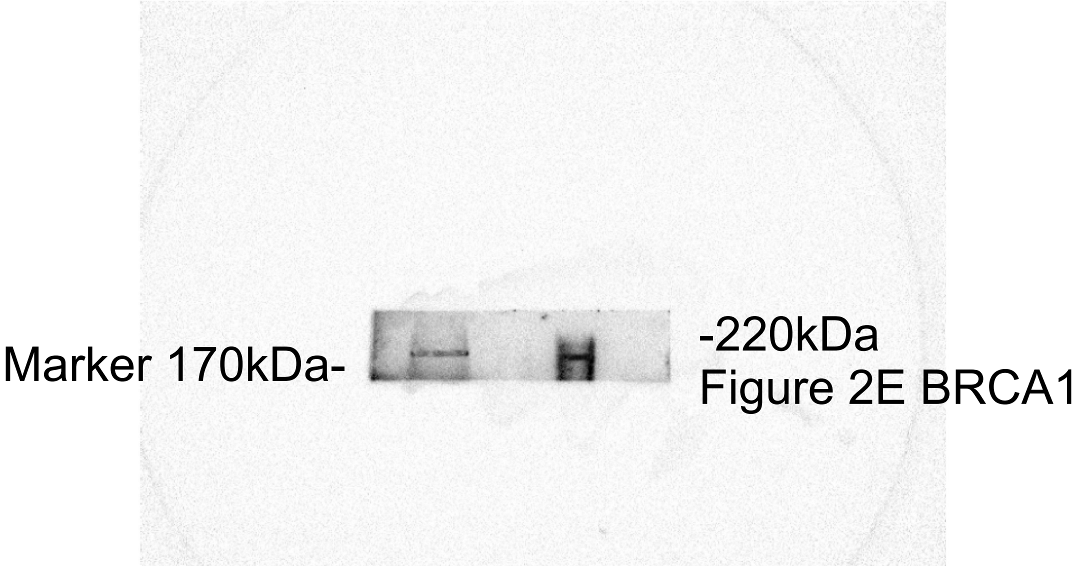
Marker 170kDa-
-220kDa
Figure 2E BRCA1

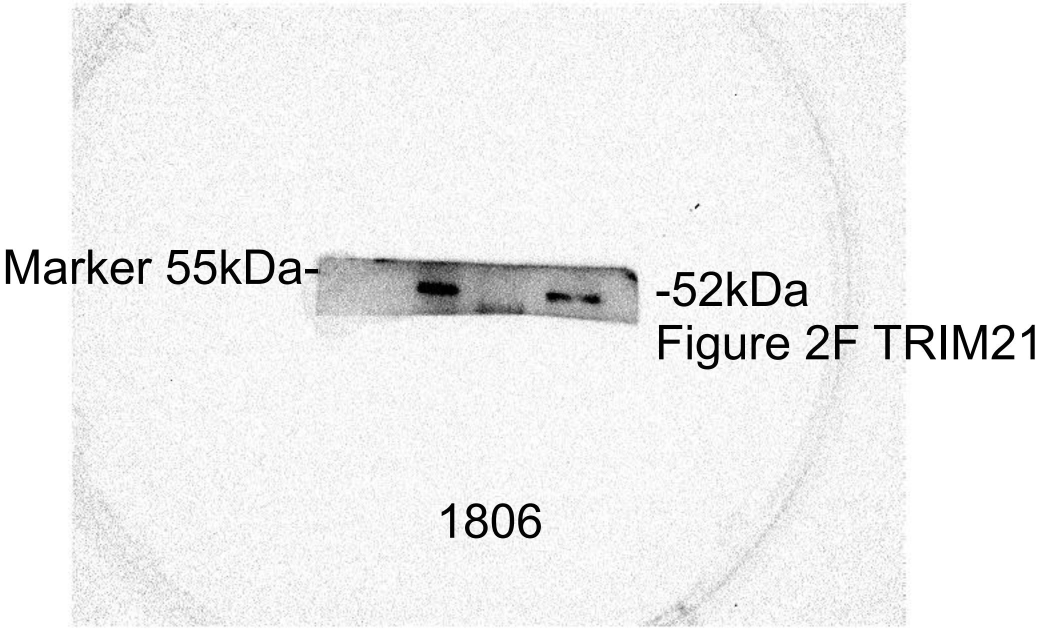
Marker 55kDa-
-52kDa
Figure 2F TRIM21
1806

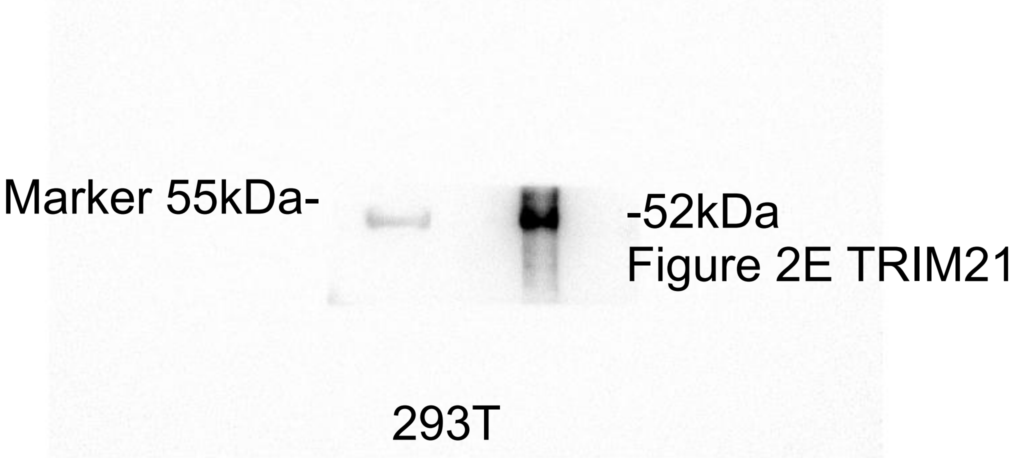
Marker 55kDa-
-52kDa
Figure 2E TRIM21
293T

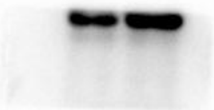

-36kDa
Figure 2F GAPDH

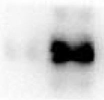

-52kDa
Figure 2F TRIM21

Marker 170kDa-

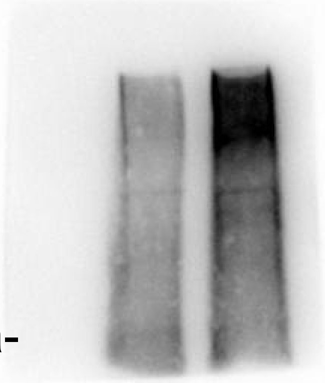

Ubiquitin

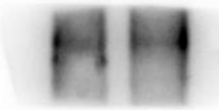

-220kDa
Figure 2F BRCA1

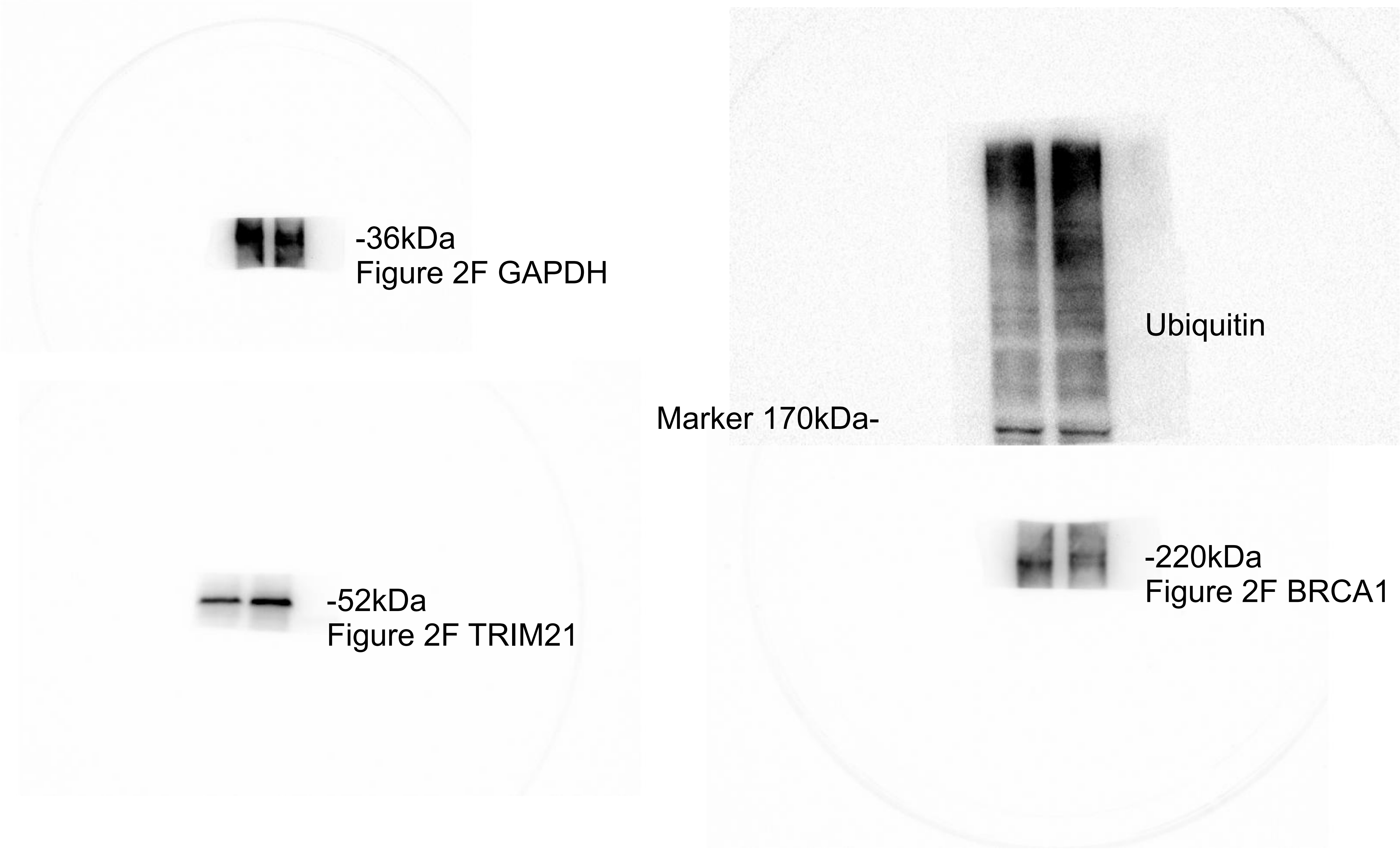
-36kDa
Figure 2F GAPDH
Ubiquitin
Marker 170kDa-
-220kDa
Figure 2F BRCA1
-52kDa
Figure 2F TRIM21

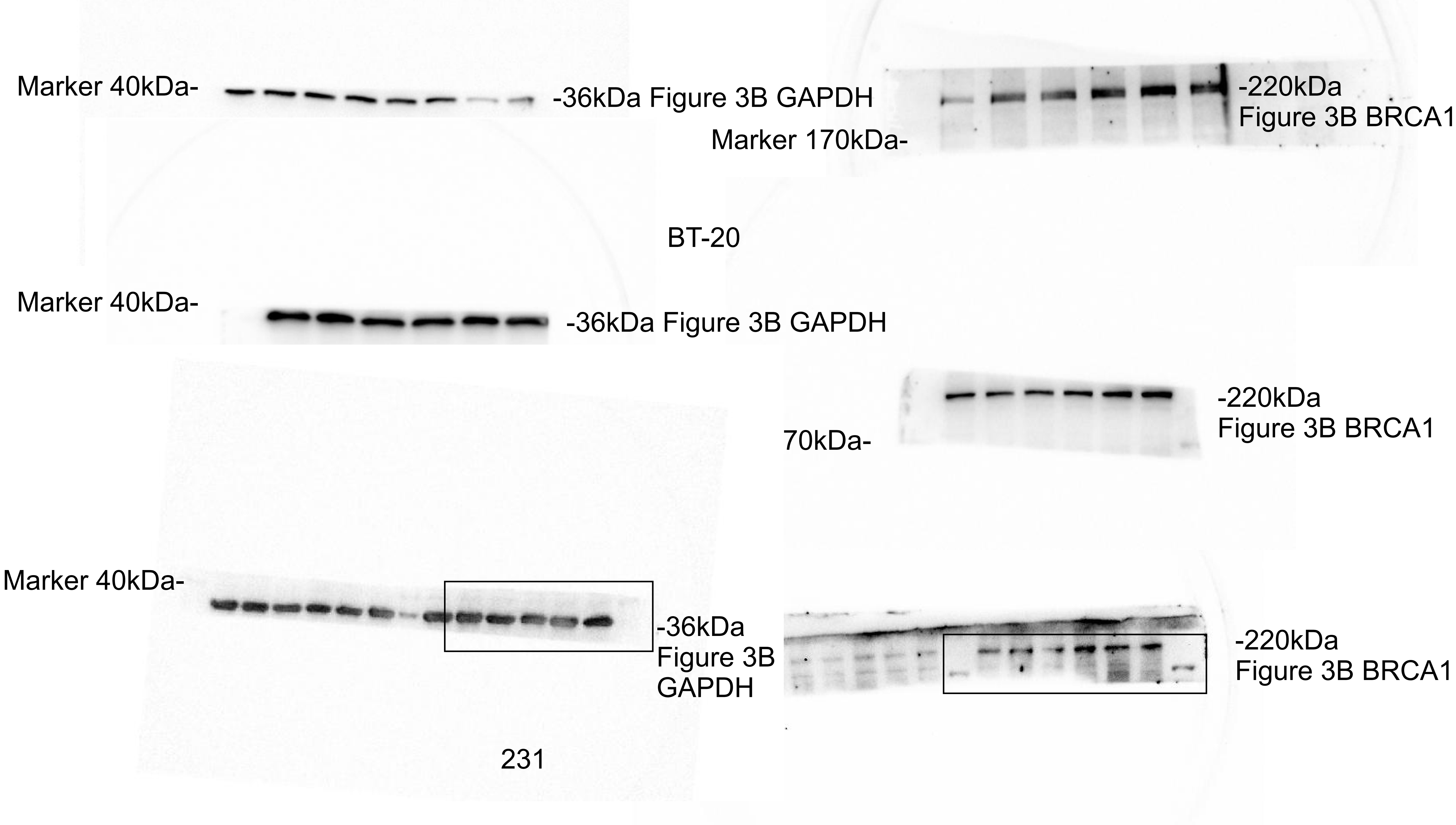
Marker 40kDa-
-36kDa Figure 3B GAPDH
-220kDa
Figure 3B BRCA1
Marker 170kDa-
BT-20
Marker 40kDa-
-36kDa Figure 3B GAPDH
-220kDa
Figure 3B BRCA1
70kDa-
Marker 40kDa-
-36kDa
Figure 3B
GAPDH
-220kDa
Figure 3B BRCA1
231

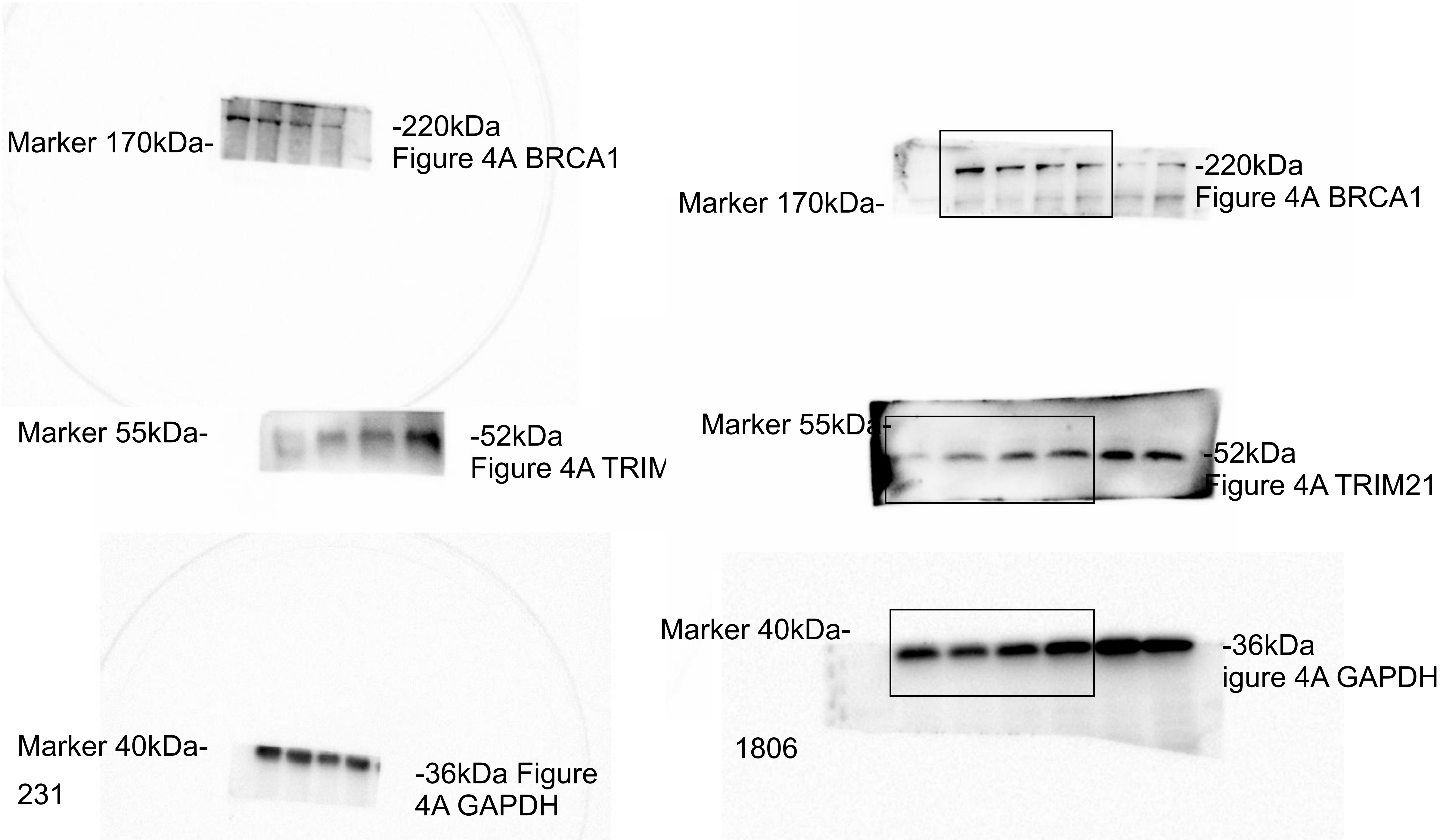
Marker 170kDa-
-220kDa
Figure 4A BRCA1
Marker 55kDa-
-52kDa
Figure 4A TRIM
Marker 40kDa-
-36kDa Figure
4A GAPDH
231
Marker 170kDa-
-220kDa
Figure 4A BRCA1
Marker 55kDa-
-52kDa
Figure 4A TRIM21
Marker 40kDa-
-36kDa
igure 4A GAPDH
1806

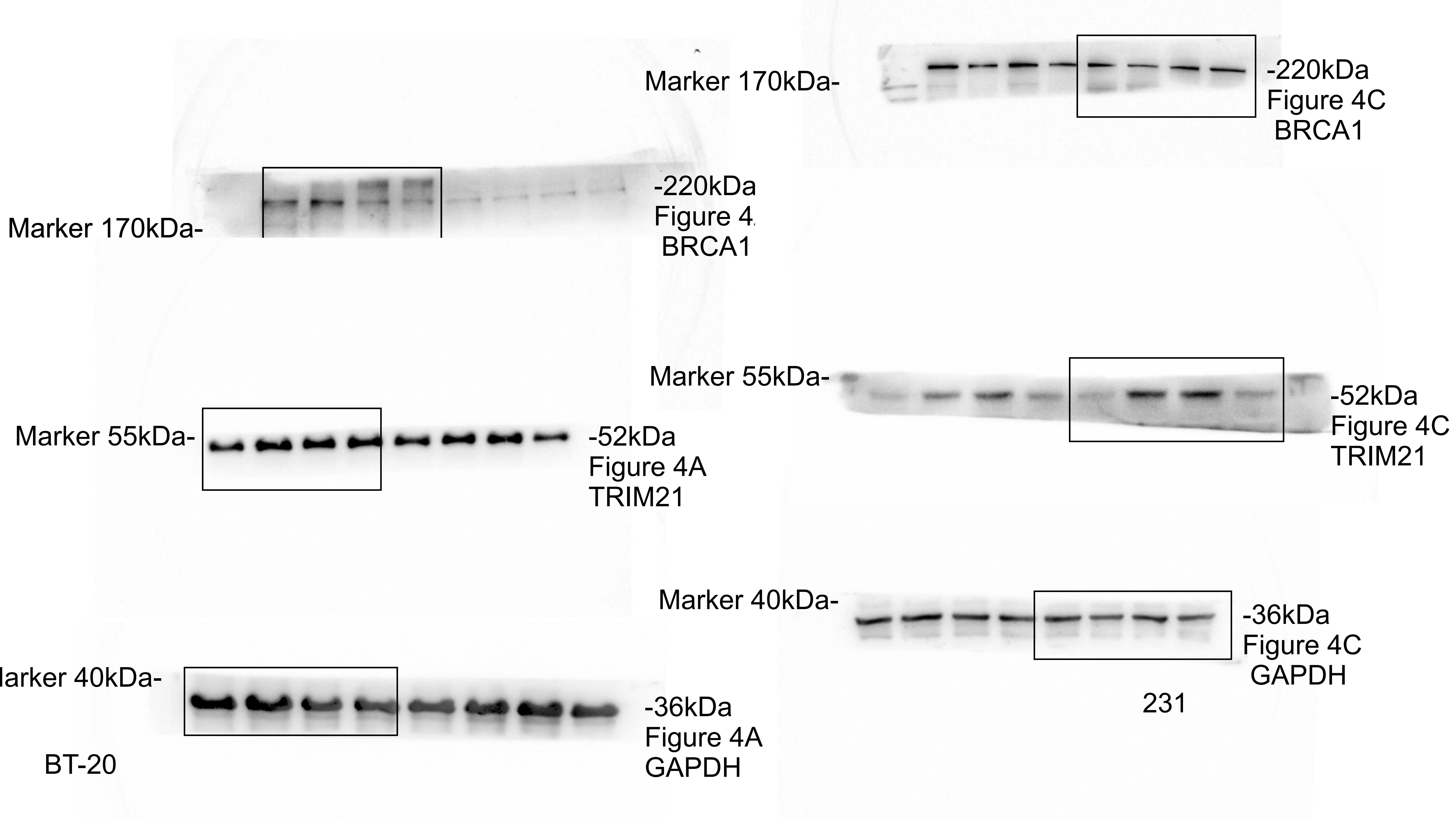
Marker 170kDa-
-220kDa
Figure 4C
BRCA1
Marker 170kDa-
-220kDa
Figure 4
BRCA1
Marker 55kDa-
-52kDa
Figure 4C
TRIM21
Marker 55kDa-
-52kDa
Figure 4A
TRIM21
Marker 40kDa-
-36kDa
Figure 4C
GAPDH
231
larker 40kDa-
-36kDa
Figure 4A
GAPDH
BT-20

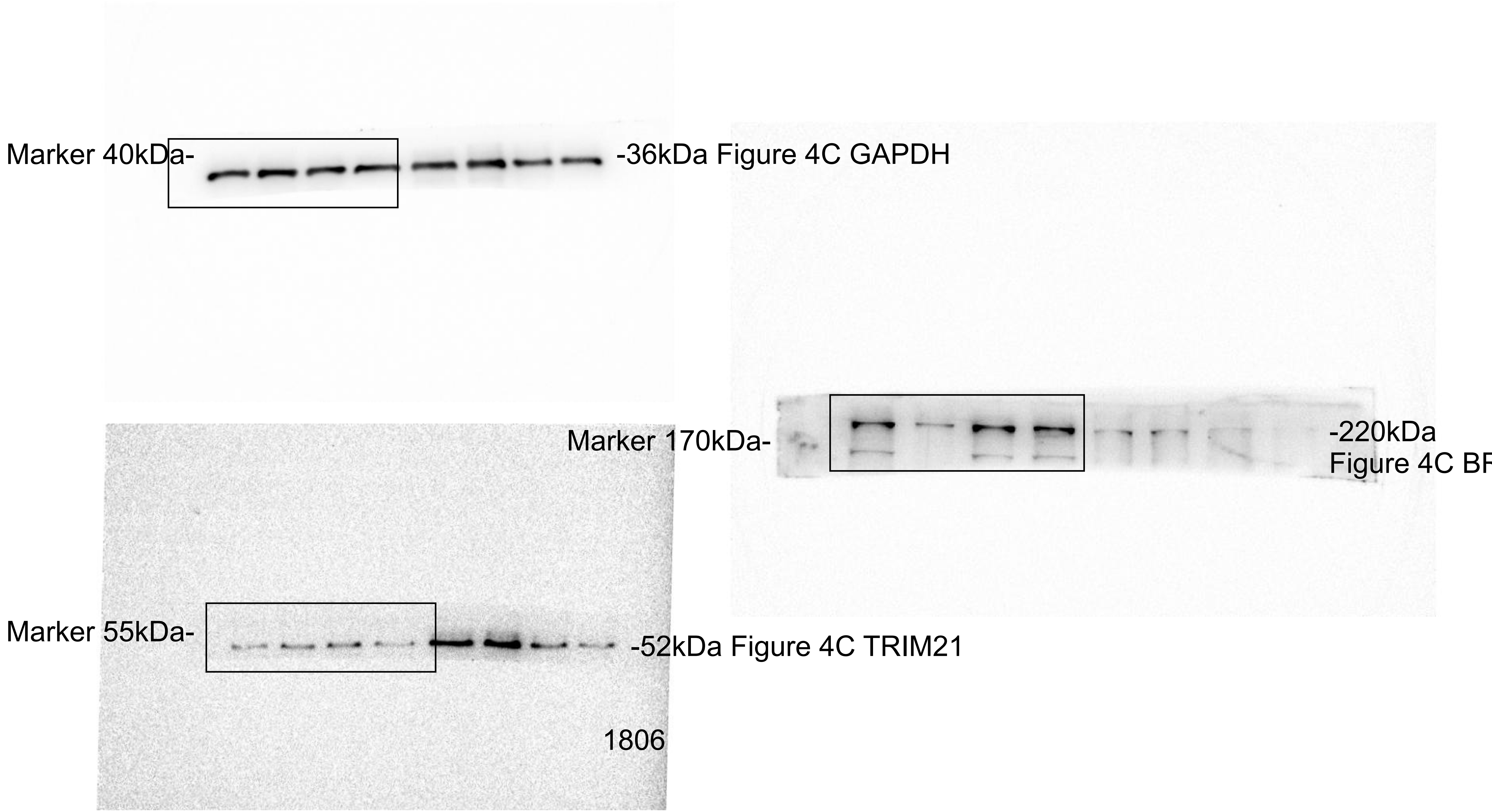

Marker 40kDa-
-36kDa Figure 4C GAPDH
Marker 170kDa-
-220kDa
Figure 4C B
Marker 55kDa-
-52kDa Figure 4C TRIM21
1806

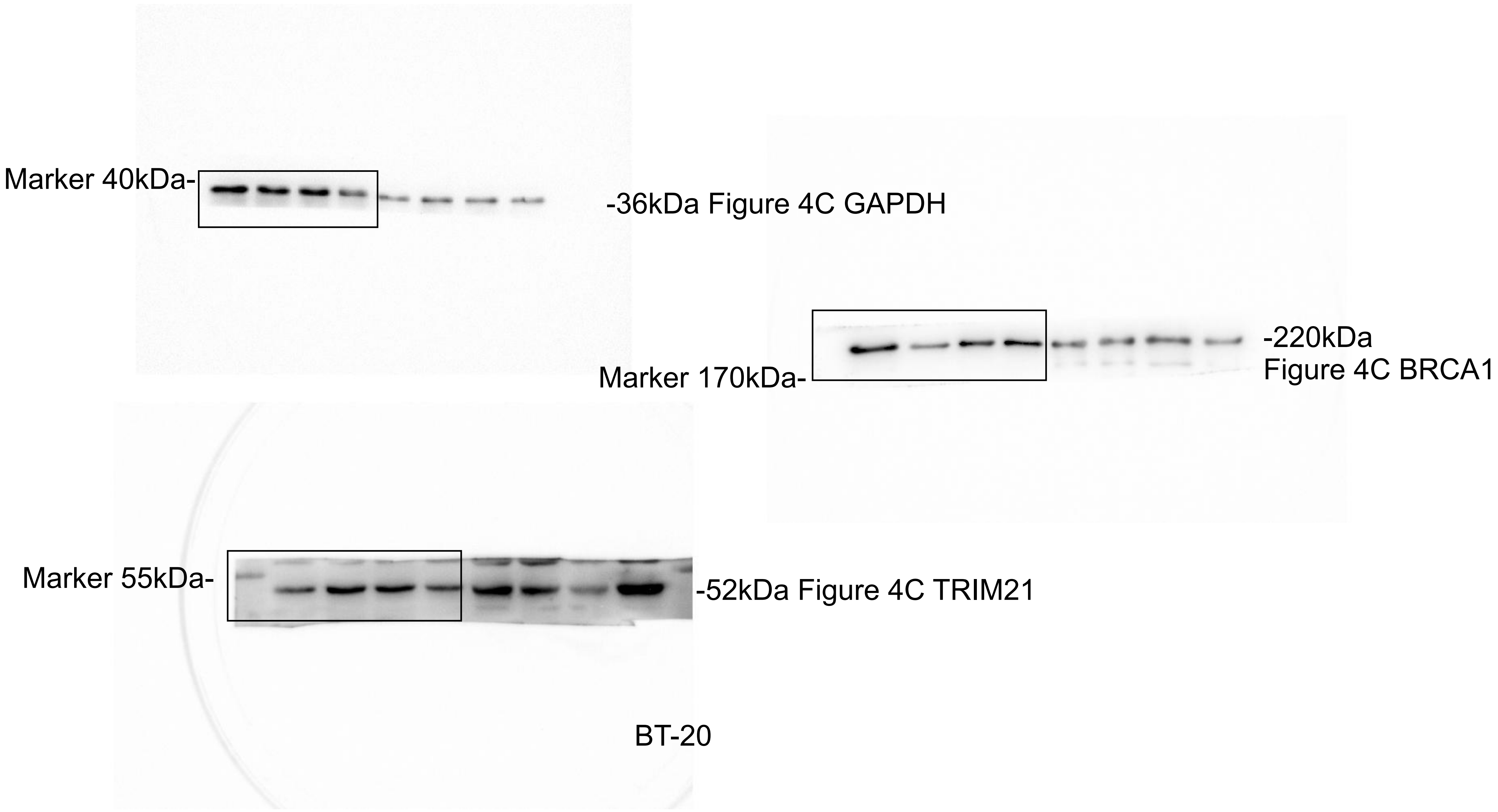
Marker 40kDa-
-36kDa Figure 4C GAPDH
-220kDa
Figure 4C BRCA1
Marker 170kDa-
Marker 55kDa-
-52kDa Figure 4C TRIM21
BT-20

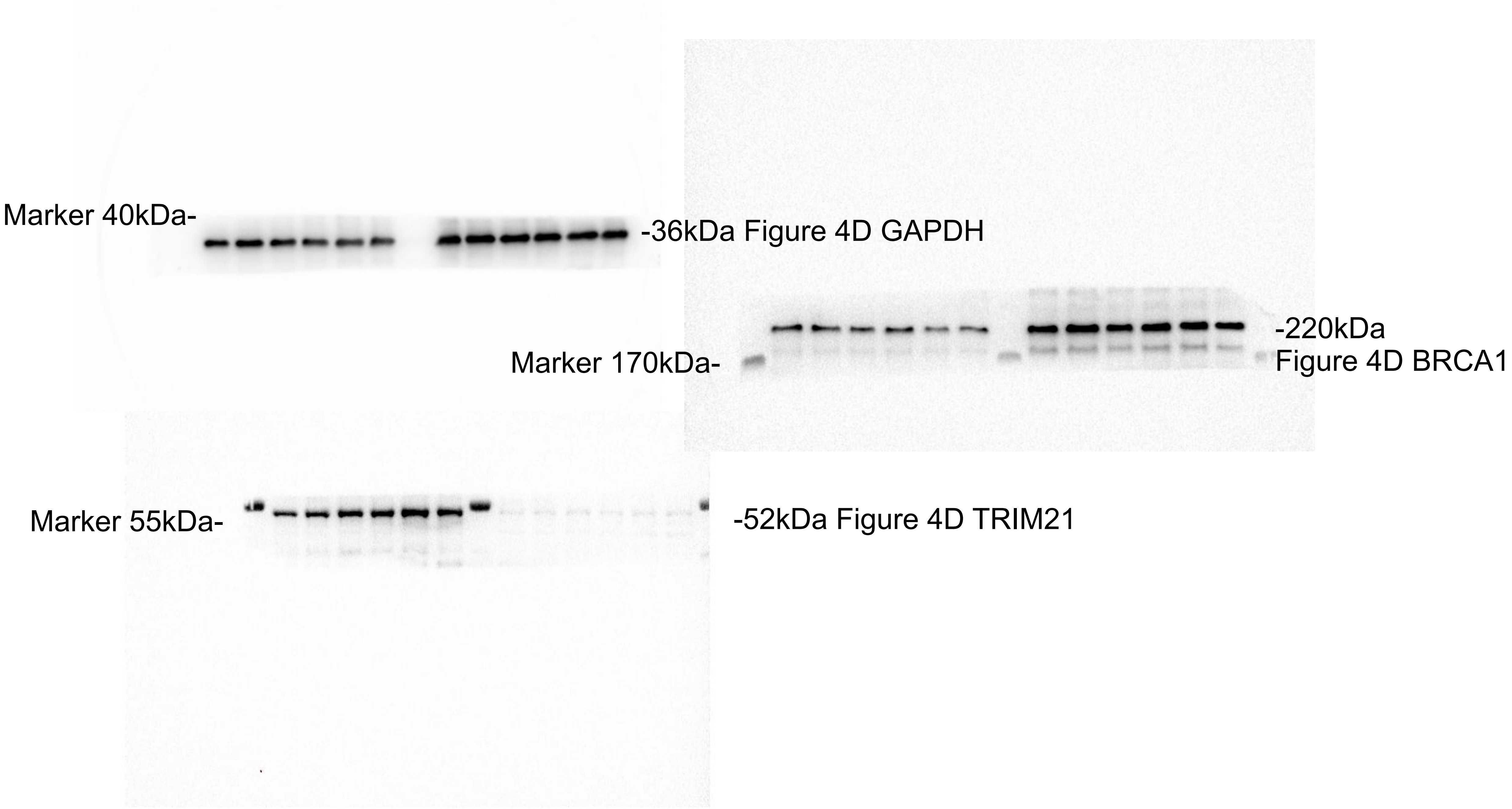
Marker 40kDa-
-36kDa Figure 4D GAPDH
-220kDa
Marker 170kDa-
Figure 4D BRCA1
Marker 55kDa-
-52kDa Figure 4D TRIM21

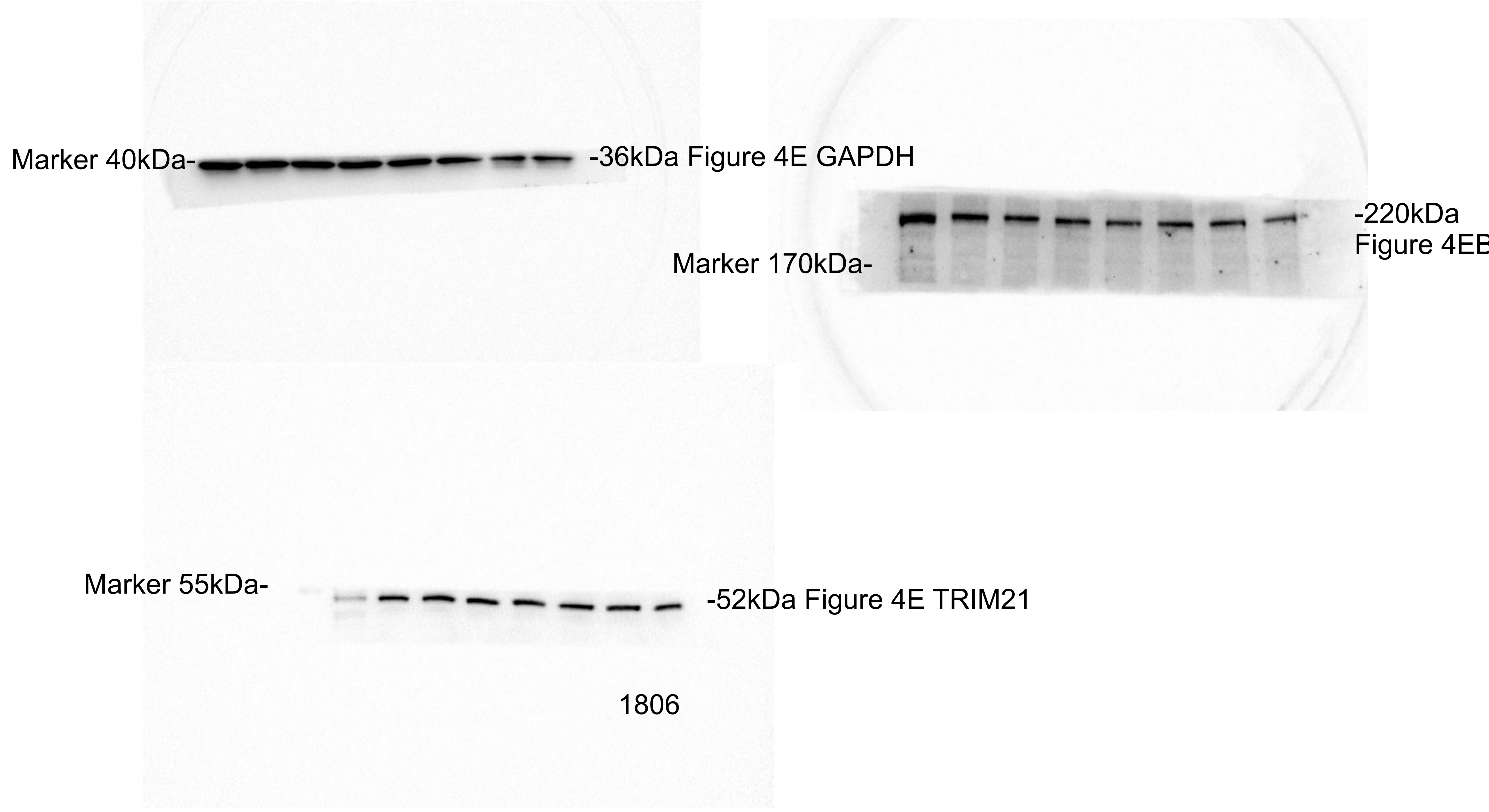
Marker 40kDa-
-36kDa Figure 4E GAPDH
-220kDa
Figure 4EB
Marker 170kDa-
Marker 55kDa-
-52kDa Figure 4E TRIM21
1806

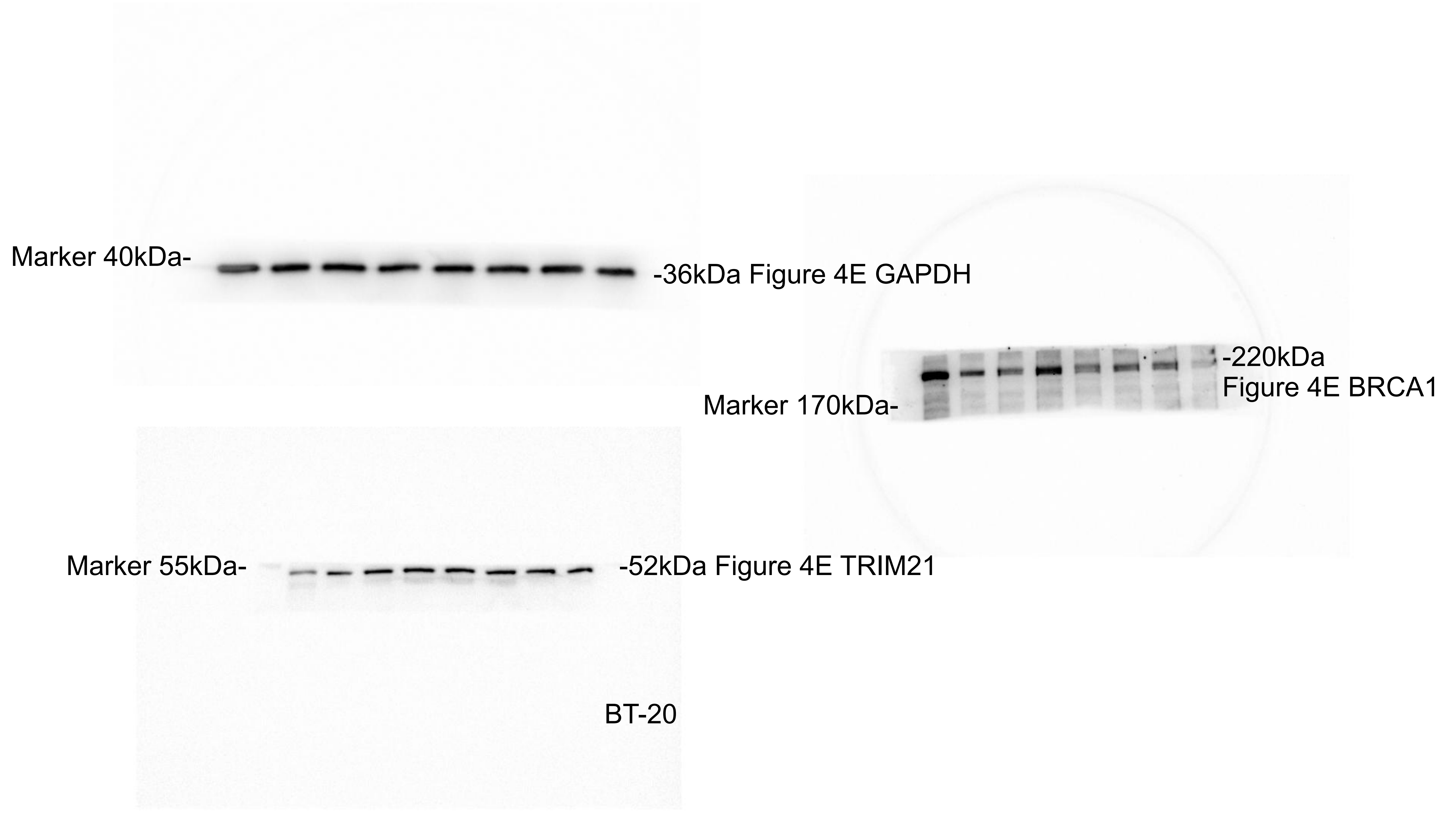
Marker 40kDa-
-36kDa Figure 4E GAPDH
-220kDa
Figure 4E BRCA1
Marker 170kDa-
Marker 55kDa-
-52kDa Figure 4E TRIM21
BT-20

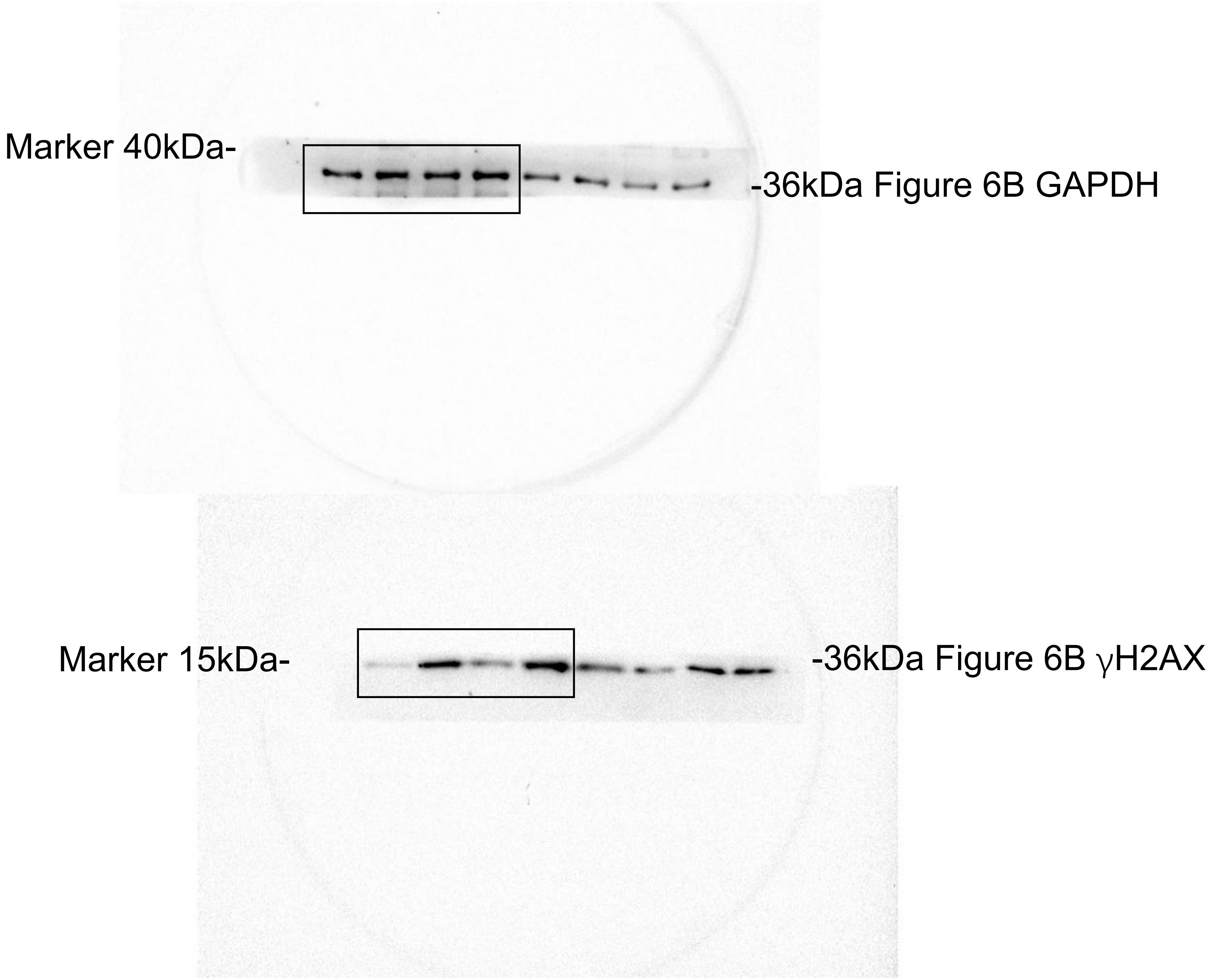
Marker 40kDa-
-36kDa Figure 6B GAPDH
Marker 15kDa-
-36kDa Figure 6B γH2AX

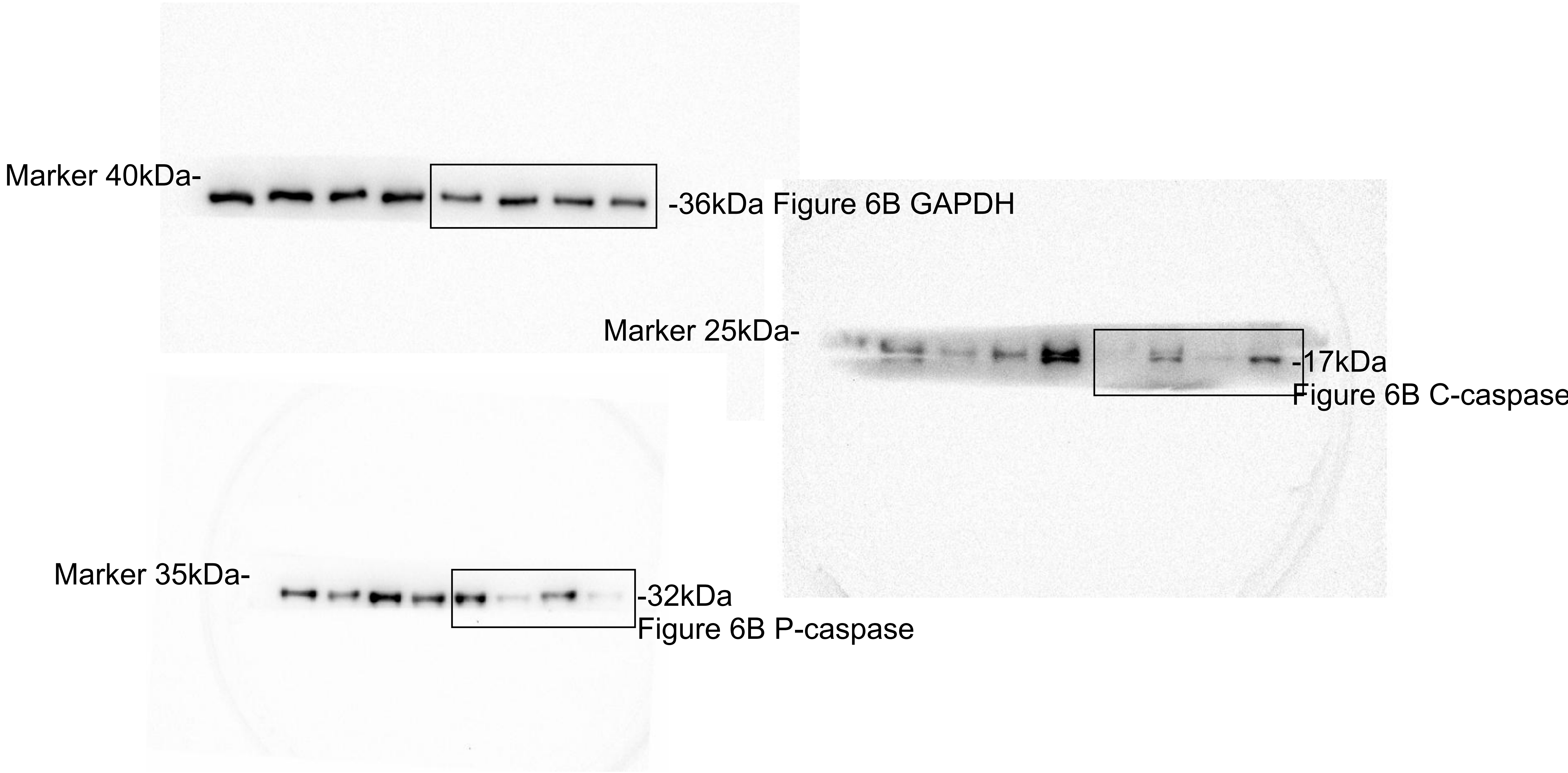
Marker 40kDa-
-36kDa Figure 6B GAPDH
Marker 25kDa-
-17kDa
Figure 6B C-caspase
Marker 35kDa-
-32kDa
Figure 6B P-caspase
